# Cardiogenic Shock With Acute Myocardial Infarction Among Older Adults in the United States

**DOI:** 10.1016/j.jacadv.2025.102078

**Published:** 2025-08-19

**Authors:** Taha Mansoor, Ali bin Abdul Jabbar, Mahmoud Ismayl, Dylan Yu, Kartik Gupta, Sachin Parikh, Austin Brubaker, Abdul Mannan Khan Minhas, Dmitry Abramov, Jagadeesh Kalavakunta, Vishal Gupta, Salim S. Virani, Santhosh K.G. Koshy

**Affiliations:** a Department of Internal Medicine, Western Michigan University Homer Stryker M.D. School of Medicine, Kalamazoo, Michigan, USA; b Department of Internal Medicine, Creighton University School of Medicine, Omaha, Nebraska, USA; c Department of Cardiology, Mayo Clinic, Rochester, Minnesota, USA; d Department of Cardiology, Henry Ford Hospital, Detroit, Michigan, USA; e Department of Biomedical Informatics, Western Michigan University Homer Stryker M.D. School of Medicine, Kalamazoo, Michigan, USA; f Section of Cardiology, Department of Medicine, Baylor College of Medicine, Houston, Texas, USA; g Department of Medicine, Baylor College of Medicine, Houston, Texas, USA; h Department of Medicine, Division of Cardiology, Loma Linda University Medical Center, Loma Linda, California, USA; i Department of Cardiology, Beacon Hospital, Kalamazoo, Michigan, USA; j Department of Medicine, Aga Khan University, Karachi, Pakistan; k Department of Population Health, Aga Khan University, Nairobi, Kenya

**Keywords:** acute myocardial infarction, cardiogenic shock, older patients

## Abstract

**Background:**

Characteristics and outcomes associated with cardiogenic shock (CS) with acute myocardial infarction (AMI) in older patients have not been well characterized.

**Objectives:**

The purpose of this study was to investigate characteristics and outcomes of older patients admitted with CS with AMI.

**Methods:**

We used the National Inpatient Sample database to evaluate clinical characteristics of hospitalizations, clinical outcomes, and health care utilization of hospitalizations of older patients ≥80 years with CS and AMI from 2003 to 2021. We also compared data from older patients with a reference relatively younger patient population (18-79 years).

**Results:**

During the study period, 207,333 hospitalizations of older patients ≥80 years and 675,491 hospitalizations of patients 18 to 79 years were identified. Between the first 4 years of the study period (2003-2006) and the final 4-year interval of the study period (2016-2019), there was an increase in the number of hospitalizations of older patients with AMI and CS (39,220-51,640). On adjusted analysis, there were increases in the percentage of older patients undergoing treatment with percutaneous coronary intervention (19.2% to 34.1%; *P* < 0.001) and encounters for palliative care (2.0% to 32.4%; *P* < 0.001) alongside a decline in in-hospital mortality (61.4% to 49.0%; *P* < 0.001) over the study period. Compared to patients 18 to 79 years of age, those aged ≥80 years had greater in-hospital mortality (51.8% vs 33.0%; *P* < 0.001).

**Conclusions:**

From 2003 to 2021, for older patients with CS and AMI, hospitalizations increased, and mortality decreased.

Cardiogenic shock (CS) is a multifactorial diagnosis that results in reduced tissue perfusion and hypoxia due to decreased cardiac output. It is most commonly caused by acute myocardial infarction (AMI) and has an adverse prognosis. Despite the high morbidity and mortality that result from CS from AMI, recent advances in treatment including revascularization strategies and temporary mechanical circulatory support (MCS) devices have resulted in outcome improvements including in older populations.^1^, ^2^, ^3^ Age plays an important part in CS prognostication and treatment selection especially as increasing age is associated with higher in-hospital mortality in CS regardless of CS severity.^3^ With increased patient survival, a greater number of older patients above 80 years of age may present with CS and older patients have been shown to have a significantly higher prevalence of CS from AMI compared with relatively younger patients.^4^ Older patients have also been mostly excluded from randomized clinical trials of CS therapy leading to a lack of data on strategies for their management.^2^ Furthermore, there remains little data regarding the outcomes of CS with AMI in older patients. In this study, we aim to use a large, nationally representative sample to study the prevalence, outcomes, and management of older patients hospitalized with CS and AMI.

## Methods

### Study data

The data for this study were obtained from January 1, 2003, to December 31, 2021, using the National Inpatient Sample database which is a part of the Healthcare Cost and Utilization Project. The National Inpatient Sample is “the largest publicly available all-payer inpatient health care database designed to produce U.S. regional and national estimates of inpatient utilization, access, cost, quality, and outcomes.^5^ Unweighted, it contains data from around 7 million hospital stays each year. Weighted, it estimates around 35 million hospitalizations nationally.” Institutional Review Board approval was not sought due to the deidentified nature and public availability of the National Inpatient Sample database.

### Study design

We conducted an observational analysis to study characteristics and outcomes of hospitalizations of older patients, designated as those ≥80 years of age, with CS and AMI. Patient hospitalizations with both a CS and AMI International Classification of Diseases (ICD) 9 or 10 code anywhere in the diagnosis fields were included in the study (Supplemental Tables 1 and 2). Data regarding demographics, clinical characteristics of hospitalizations, insurance type, ZIP code income quartile, hospital characteristics, region, and outcomes, including mortality were extracted. We also obtained these data for relatively younger patients 18 to 79 years of age during the same period (January 1, 2003, to December 31, 2021) to provide a comparison group for the older population.

### Outcomes

We analyzed outcomes including in-hospital mortality, length of hospital stays, cost of hospitalization, discharge disposition, cardiac arrest, cardiac tamponade, pericarditis, acute kidney injury, acute posthemorrhagic anemia, gastrointestinal bleeding, pulmonary embolism, respiratory failure, encounters for palliative care, and do not resuscitate (DNR) orders. We also investigated the use of invasive mechanical ventilation, temporary MCS (percutaneous left ventricular assist devices [pLVAD], extracorporeal membrane oxygenation [ECMO], intra-aortic balloon pumps [IABP]), and renal replacement therapy. Lastly, we compared the performance of percutaneous coronary intervention (PCI), coronary artery bypass grafting (CABG), gastrostomy, and tracheostomy.

### Statistical analysis

Descriptive statistics were collected on all variables of interest based on 4 groups of 4 years: 2003 to 2006, 2007 to 2010, 2011 to 2014, 2016 to 2019, and 1 group of 2 years 2020 to 2021. 2020 and 2021 were analyzed separately due to the potential skewing of results from significant burdens placed on inpatient facilities from the COVID-19 pandemic. We excluded 2015 from the analysis due to the transition of ICD-9 to ICD-10 codes this year. We used SAS Studio version 3.82 or SAS Propriety Software version 9.4 (SAS Institute) for all analyses using the survey commands in SAS, and updated trend weights were used for all years of the analysis. Weighted frequencies and percentages were reported based on an individual’s age group (≥80 vs 18-79 years).

For clinical characteristics of hospitalizations, analysis of variance tests were run to see if there were statistically significant differences between numeric variables. Chi-square tests were run to see if there was a difference in clinical characteristics of hospitalizations, for categorical variables by time period. For outcomes, multivariable regression was used to attempt to reduce confounding. For categorical variables, multivariable logistic regression was used whereas for numeric variables, multivariable linear regression was used. All clinical characteristics of hospitalizations were considered for the model, with variables being removed if violations of multicollinearity were present. *P* values for all outcomes were derived from multivariable regression analysis to attempt to reduce confounding. *P* values for the time period based on each regression model were documented by outcome and age group. In addition, the difference in outcome between age groups was tested under the same multivariable regression approach, with *P* values representing the difference in age group.

We inflated total hospitalization costs to 2021 U.S. dollars using the Consumer Price Index inflation calculator published by the U.S. Bureau of Labor Statistics.^6^ We calculated hospitalizations with CS and AMI per 100,000 U.S. adults aged ≥80 and 18 to 79 years, for which the denominator was extracted from the U.S. Census Bureau estimates of the U.S. resident population of adults aged ≥80 years and 18 to 79 years old for each study year.^7^^,^^8^ Throughout the analysis, adherence was maintained to the research methodological standards of the National Inpatient Sample database (Central Illustration).^9^

**Central Illustration:**
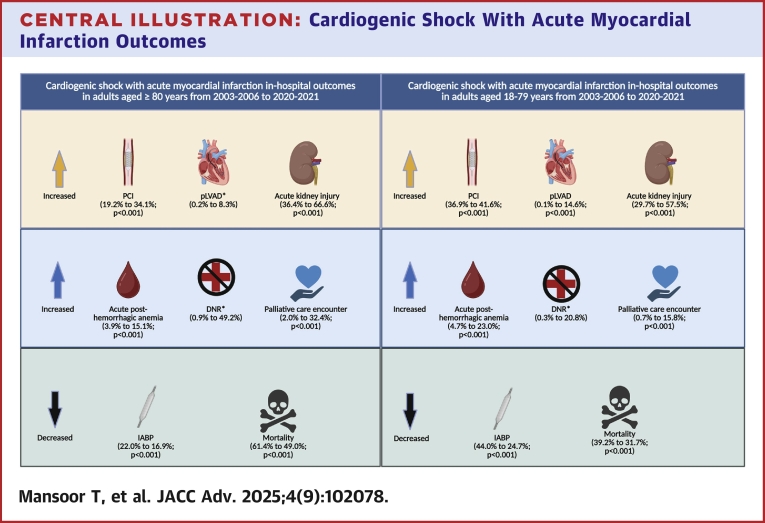
**Cardiogenic Shock With Acute Myocardial Infarction Outcomes** Outcomes of cardiogenic shock and acute myocardial infarction in adults aged ≥80 years and 18 to 79 years in the United States. ∗Figures are quoted from 2007-2010 to 2020-2021 due to noreportable data in 2003-2006. Created in BioRender. Mansoor T (2025), https://BioRender.com/2u379tq. DNR = do not resuscitate; IABP = intra-aortic balloon pump; PCI = percutaneous coronary intervention; pLVAD = percutaneous left ventricular assist devices.

## Results

### Older patient hospitalizations

#### Clinical characteristics of hospitalizations

**Figure 1:**
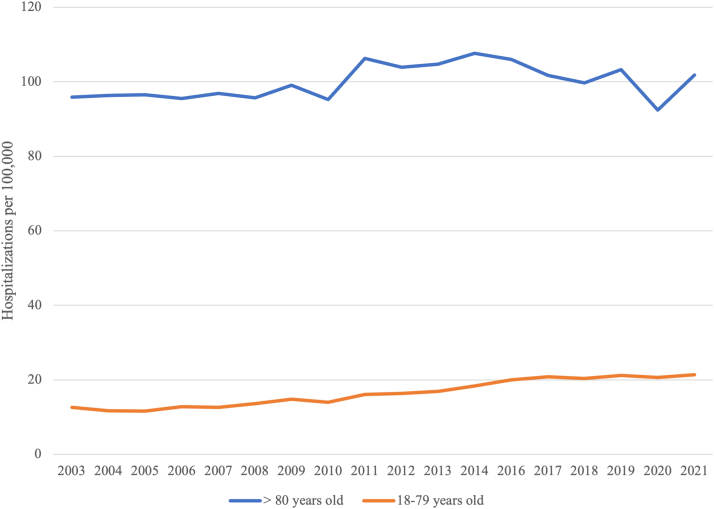
**Cardiogenic Shock With Acute Myocardial Infarction Hospitalization** Comparison of hospitalizations with cardiogenic shock and acute myocardial infarction per 100,000 adults ≥80 years and 18 to 79 years in the United States from 2003 to 2021.

From 2003 to 2021, a total of 207,333 hospitalizations of older patients with a diagnosis of CS and AMI were identified, and older patients accounted for 23.5% of all adult hospitalizations for CS and AMI during the entire study period. The median age in older patients was 84 years, 50.2% were female, and 80.0% were White. Most older patients were beneficiaries of Medicare (91.7%) and admitted to urban teaching hospitals (57.7%). Between the first 4 years of the study period (2003-2006) and the final 4-year interval of the study (2016-2019), there was an increase in the number of hospitalizations of older patients (39,220-51,640). The number of older patient hospitalizations per 100,000 of the older patient population also had a general increase over the study time period (Figure 1). The proportion of CS among all hospitalizations with AMI for patients ≥80 years of age increased from 3.9% to 7.0% (*P* < 0.001). The percentage of older patients accounting for all adult hospitalizations with CS and AMI decreased consistently over the study period from 27.7% (2003-2006) to 24.1% (2011-2014) to 19.4% (2020-2021). In addition, most hospitalizations occurred in the Southern region (34.4%). The most observed comorbidities in older patients were congestive heart failure (66.0%), hypertension (57.7%), atrial fibrillation (38.7%), dyslipidemia (39.2%), and renal failure (33.4%) (Table 1, Table 2).

**Table 1:** Temporal Changes in Clinical Characteristics of Hospitalizations With Cardiogenic Shock and Acute Myocardial Infarction of Patients Aged ≥80 Years in the United States, 2003-2021

	2003-2006	2007-2010	2011-2014	2016-2019	2020-2021	Total	*P* Value
Sample size	39,220 (18.9%)	42,539 (20.5%)	49,248 (23.8%)	51,640 (24.9%)	24,685 (11.9%)	207,333	
Total hospitalizations with acute myocardial infarction	998,243 (23.9%)	980,469 (23.4%)	950,454 (22.7%)	899,730 (21.5%)	354,290 (8.5%)	4,183,184	
Proportion of cardiogenic shock among all hospitalizations with acute myocardial infarction	3.9%	4.3%	5.2%	5.7%	7.0%	5.0%	<0.001
Age, y (median, IQR)	84 (81-87)	84 (82-88)	85 (82-88)	84 (82-88)	84 (81-88)	84 (82-88)	<0.001
Sex							<0.001
Female	21,280 (54.3%)	22,298 (52.4%)	24,729 (50.2%)	24,585 (47.6%)	11,200 (45.4%)	104,093 (50.2%)	
Male	17,935 (45.7%)	20,241 (47.6%)	24,514 (49.8%)	27,055 (52.4%)	13,480 (54.6%)	103,225 (49.8%)	
Race/ethnicity							<0.001
White	25,127 (84.6%)	28,994 (81.7%)	36,973 (80.5%)	38,395 (77.2%)	18,315 (76.6%)	147,805 (80.0%)	
Black	1,331 (4.5%)	1,914 (5.4%)	2,917 (6.4%)	3,500 (7.0%)	1,710 (7.2%)	11,373 (6.2%)	
Hispanic	1,753 (5.9%)	2,041 (5.8%)	2,965 (6.5%)	3,845 (7.7%)	1,940 (8.1%)	12,543 (6.8%)	
Asian or Pacific Islander	785 (2.6%)	1,192 (3.4%)	1,488 (3.2%)	2,060 (4.1%)	1,125 (4.7%)	6,649 (3.6%)	
Native American/Other	724 (2.4%)	1,361 (3.8%)	1,595 (3.5%)	1,915 (3.9%)	825 (3.5%)	6,420 (3.5%)	
Insurance type							<0.001
Medicare	7,707 (92.8%)	38,736 (91.1%)	45,275 (92.1%)	47,530 (92.1%)	22,070 (89.5%)	189,986 (91.7%)	
Medicaid	349 (0.9%)	496 (1.2%)	531 (1.1%)	615 (1.2%)	460 (1.9%)	2,451 (1.2%)	
Private insurance	1,950 (5.0%)	2,587 (6.1%)	2,461 (5.0%)	2,400 (4.7%)	1,355 (5.5%)	10,753 (5.2%)	
Self-pay	217 (0.6%)	268 (0.6%)	310 (0.6%)	325 (0.6%)	205 (0.8%)	1,325 (0.6%)	
No charge/other	300 (0.8%)	419 (1.0%)	568 (1.2%)	740 (1.4%)	565 (2.3%)	2,592 (1.3%)	
Median household income quartile of patient’s zip code							0.442
0-25th	9,481 (24.7%)	9,760 (23.4%)	12,248 (25.4%)	13,015 (25.6%)	5,830 (23.9%)	50,335 (24.7%)	
26-50th	9,947 (25.9%)	11,475 (27.5%)	12,385 (25.6%)	13,030 (25.6%)	6,790 (27.9%)	53,627 (26.3%)	
50-75th	9,553 (24.9%)	10,398 (24.9%)	12,054 (25.0%)	12,785 (25.1%)	6,005 (24.7%)	50,796 (24.9%)	
75-100th	9,390 (24.5%)	10,117 (24.2%)	11,622 (24.1%)	12,060 (23.7%)	5,725 (23.5%)	48,914 (24.0%)	
Hospital location							<0.001
Rural	4,536 (11.6%)	4,156 (9.8%)	3,956 (8.1%)	2,990 (5.8%)	1,380 (5.6%)	17,018 (8.2%)	
Urban nonteaching	18,132 (46.2%)	19,257 (45.6%)	18,062 (36.8%)	10,870 (21.1%)	4,235 (17.2%)	70,556 (34.1%)	
Urban teaching	16,542 (42.2%)	18,815 (44.6%)	27,126 (55.2%)	37,780 (73.2%)	19,070 (77.3%)	119,332 (57.7%)	
Region of hospital							0.039
Northeast	9,396 (24.0%)	9,136 (21.5%)	10,998 (22.3%)	10,130 (19.6%)	4,660 (18.9%)	44,320 (21.4%)	
Midwest	9,008 (23.0%)	10,428 (24.5%)	10,664 (21.7%)	11,575 (22.4%)	5,475 (22.2%)	47,149 (22.7%)	
South	13,267 (33.8%)	13,778 (32.4%)	17,099 (34.7%)	18,230 (35.3%)	8,870 (35.9%)	71,244 (34.4%)	
West	7,550 (19.3%)	9,198 (21.6%)	10,487 (21.3%)	11,705 (22.7%)	5,680 (23.0%)	44,620 (21.5%)	
Comorbidities							
Congestive heart failure	25,731 (65.6%)	26,357 (62.0%)	31,613 (64.2%)	35,570 (68.9%)	17,650 (71.5%)	136,920 (66.0%)	<0.001
Valvular disease	1,803 (4.7%)	1,985 (6.2%)	3,445 (7.0%)	15,320 (29.7%)	6,780 (27.5%)	29,333 (15.0%)	<0.001
Chronic pulmonary disease	7,332 (19.0%)	6,427 (20.2%)	10,123 (20.6%)	8,590 (16.6%)	1,735 (7.0%)	34,208 (17.5%)	<0.001
Obesity	439 (1.1%)	853 (2.7%)	2,371 (4.8%)	2,195 (4.3%)	505 (2.1%)	6,363 (3.3%)	<0.001
Dementia	1,317 (3.4%)	2,009 (4.7%)	2,391 (4.9%)	8,575 (16.6%)	3,900 (15.8%)	18,192 (8.8%)	<0.001
Dyslipidemia	5,278 (13.5%)	12,542 (29.5%)	21,352 (43.4%)	27,695 (53.6%)	14,430 (58.5%)	81,297 (39.2%)	<0.001
Iron deficiency	1,214 (3.1%)	1,558 (3.7%)	1,836 (3.7%)	2,440 (4.7%)	1,010 (4.1%)	8,058 (3.9%)	<0.001
Diabetes mellitus	7,929 (20.6%)	8,442 (26.5%)	15,590 (31.7%)	15,080 (29.2%)	3,335 (13.5%)	50,376 (25.7%)	<0.001
Hypertension	16,855 (43.7%)	17,940 (56.3%)	33,059 (67.1%)	37,065 (71.8%)	8,085 (32.8%)	113,003 (57.7%)	<0.001
Liver disease	226 (0.6%)	264 (0.8%)	630 (1.3%)	1,990 (3.9%)	1,140 (4.6%)	4,251 (2.2%)	<0.001
Neurological disorders	2,902 (7.5%)	2,913 (9.1%)	5,115 (10.4%)	10,630 (20.6%)	5,790 (23.5%)	27,350 (14.0%)	<0.001
Peripheral vascular disease	3,369 (8.7%)	4,060 (12.7%)	8,078 (16.4%)	5,625 (10.9%)	1,100 (4.5%)	22,233 (11.3%)	<0.001
Renal failure	6,860 (17.8%)	9,102 (28.5%)	17,911 (36.4%)	21,025 (40.7%)	10,565 (42.8%)	65,462 (33.4%)	<0.001
Malnutrition	1,537 (3.9%)	2,708 (6.4%)	4,650 (9.4%)	4,905 (9.5%)	2,395 (9.7%)	16,195 (7.8%)	<0.001
Atrial fibrillation	13,520 (34.5%)	13,774 (32.4%)	18,982 (38.5%)	22,885 (44.3%)	11,060 (44.8%)	80,220 (38.7%)	<0.001
History of malignancy	1,630 (4.2%)	2,980 (7.0%)	4,882 (9.9%)	6,350 (12.3%)	2,785 (11.3%)	18,627 (9.0%)	<0.001
Coagulopathy	3,358 (8.7%)	3,457 (10.8%)	7,718 (15.7%)	9,200 (17.8%)	4,385 (17.7%)	28,118 (14.3%)	<0.001
Solid tumor without metastasis	722 (1.9%)	709 (2.2%)	1,152 (2.3%)	1,270 (2.5%)	250 (1.0%)	4,102 (2.1%)	<0.001
Drug abuse	NR^a^	NR^a^	64 (0.1%)	85 (0.2%)	NR^a^	NR^a^	-
Alcohol abuse	205 (0.5%)	231 (0.7%)	433 (0.9%)	300 (0.6%)	80 (0.3%)	1,249 (0.6%)	0.001
Nicotine dependence	1,680 (4.3%)	3,938 (9.3%)	7,988 (16.2%)	2,225 (4.3%)	1,070 (4.3%)	16,900 (8.2%)	<0.001
Deficiency anemia	5,219 (13.5%)	7,248 (22.7%)	13,795 (28.0%)	14,520 (28.1%)	6,925 (28.1%)	47,707 (24.3%)	<0.001
Hypothyroidism	3,169 (8.2%)	3,907 (12.3%)	8,256 (16.8%)	6,625 (12.8%)	1,305 (5.3%)	23,261 (11.9%)	<0.001
Previous PCI	971 (2.5%)	2,375 (5.6%)	4,104 (8.3%)	6,065 (11.7%)	2,730 (11.1%)	16,246 (7.8%)	<0.001
Previous CABG	2,393 (6.1%)	3,778 (8.9%)	5,620 (11.4%)	5,735 (11.1%)	2,430 (9.8%)	19,955 (9.6%)	<0.001
Previous MI	2,115 (5.4%)	3,219 (7.6%)	5,164 (10.5%)	7,015 (13.6%)	3,055 (12.4%)	20,568 (9.9%)	<0.001
Cerebrovascular disease	1,765 (4.5%)	4,058 (9.5%)	6,506 (13.2%)	7,970 (15.4%)	3,620 (14.7%)	23,918 (11.5%)	<0.001
Type of myocardial infarction							
STEMI	24,334 (62.0%)	22,459 (52.8%)	21,381 (43.4%)	20,260 (39.2%)	9,455 (38.3%)	97,889 (47.2%)	<0.001
NSTEMI	14,886 (38.0%)	20,080 (47.2%)	27,867 (56.6%)	31,380 (60.8%)	15,230 (61.7%)	109,444 (52.8%)	<0.001

Values are n (%) unless otherwise indicated.

CABG = coronary artery bypass grafting; MI = myocardial infarction; NSTEMI = non-ST-elevation myocardial infarction; PCI = percutaneous coronary intervention; STEMI = ST-elevation myocardial infarction.

a n < 11 data are not reported (NR) according to HCUP recommendations. In some cases, additional suppression is applied to prevent deductive disclosure.

**Table 2:** Clinical Characteristics of Hospitalizations With Cardiogenic Shock and Acute Myocardial Infarction of Patients Aged 18-79 vs ≥ 80 Years in the United States, 2003-2021

	Age 18-79 Years	Age ≥80 Years	Total	*P* Value
Sample size	675,491	207,333	882,824	
Total hospitalizations with acute myocardial infarction	11,716,030	4,183,184	15,899,214	
Proportion of cardiogenic shock among all hospitalizations with acute myocardial infarction	5.8%	5.0%	5.6%	<0.001
Age, y (median, IQR)	65 (57-72)	84 (82-88)	69 (60-78)	<0.001
Sex				<0.001
Female	231,699 (34.3%)	104,093 (50.2%)	335,792 (38.0%)	
Male	443,745 (65.7%)	103,225 (49.8%)	546,970 (62.0%)	
Race/ethnicity				<0.001
White	433,645 (72.2%)	147,805 (80.0%)	581,450 (74.0%)	
Black	60,221 (10.0%)	11,373 (6.2%)	71,594 (9.1%)	
Hispanic	56,238 (9.4%)	12,543 (6.8%)	68,781 (8.8%)	
Asian or Pacific Islander	21,135 (3.5%)	6,649 (3.6%)	27,784 (3.5%)	
Native American/Other	29,310 (4.9%)	6,420 (3.5%)	35,730 (4.5%)	
Insurance type				<0.001
Medicare	361,689 (53.6%)	189,986 (91.7%)	551,675 (62.6%)	
Medicaid	68,393 (10.1%)	2,451 (1.2%)	70,844 (8.0%)	
Private insurance	179,997 (26.7%)	10,753 (5.2%)	190,750 (21.6%)	
Self-pay	39,525 (5.9%)	1,325 (0.6%)	40,850 (4.6%)	
No charge/other	24,722 (3.7%)	2,592 (1.3%)	27,314 (3.1%)	
Median household income quartile of patient’s zip code				<0.001
0-25th	195,607 (29.7%)	50,335 (24.7%)	245,942 (28.5%)	
26-50th	178,563 (27.1%)	53,627 (26.3%)	232,190 (26.9%)	
50-75th	157,057 (23.8%)	50,796 (24.9%)	207,853 (24.1%)	
75-100th	127,702 (19.4%)	48,914 (24.0%)	176,616 (20.5%)	
Hospital location				<0.001
Rural	36,181 (5.4%)	17,018 (8.2%)	53,199 (6.0%)	
Urban nonteaching	194,005 (28.8%)	70,556 (34.1%)	264,561 (30.0%)	
Urban teaching	443,973 (65.9%)	119,332 (57.7%)	563,305 (63.9%)	
Region of hospital				<0.001
Northeast	110,383 (16.3%)	44,320 (21.4%)	154,703 (17.5%)	
Midwest	149,366 (22.1%)	47,149 (22.7%)	196,515 (22.3%)	
South	272,382 (40.3%)	71,244 (34.4%)	343,626 (38.9%)	
West	143,360 (21.2%)	44,620 (21.5%)	187,980 (21.3%)	
Comorbidities				
Congestive heart failure	399,401 (59.1%)	136,920 (66.0%)	536,321 (60.8%)	<0.001
Valvular disease	64,734 (10.1%)	29,333 (15.0%)	94,067 (11.2%)	<0.001
Chronic pulmonary disease	127,121 (19.8%)	34,208 (17.5%)	161,329 (19.2%)	<0.001
Obesity	73,676 (11.5%)	6,363 (3.3%)	80,039 (9.5%)	<0.001
Dementia	10,561 (1.6%)	18,192 (8.8%)	28,753 (3.3%)	<0.001
Dyslipidemia	284,389 (42.1%)	81,297 (39.2%)	365,686 (41.4%)	<0.001
Iron deficiency	20,893 (3.1%)	8,058 (3.9%)	28,951 (3.3%)	<0.001
Diabetes mellitus	201,321 (31.3%)	50,376 (25.7%)	251,697 (30.0%)	<0.001
Hypertension	322,805 (50.2%)	113,003 (57.7%)	435,808 (52.0%)	<0.001
Liver disease	28,800 (4.5%)	4,251 (2.2%)	33,051 (3.9%)	<0.001
Neurological disorders	83,995 (13.1%)	27,350 (14.0%)	111,345 (13.3%)	<0.001
Peripheral vascular disease	64,881 (10.1%)	22,233 (11.3%)	87,114 (10.4%)	<0.001
Renal failure	150,330 (23.4%)	65,462 (33.4%)	215,792 (25.7%)	<0.001
Malnutrition	53,722 (8.0%)	16,195 (7.8%)	69,917 (7.9%)	0.387
Atrial fibrillation	165,219 (24.5%)	80,220 (38.7%)	245,439 (27.8%)	<0.001
History of malignancy	30,728 (4.6%)	18,627 (9.0%)	49,355 (5.6%)	<0.001
Coagulopathy	123,071 (19.2%)	28,118 (14.3%)	151,189 (18.0%)	<0.001
Solid tumor without metastasis	9,600 (1.5%)	4,102 (2.1%)	13,702 (1.6%)	<0.001
Drug abuse	14,960 (2.3%)	NR^a^	NR^a^	-
Alcohol abuse	24,876 (3.9%)	1,249 (0.6%)	26,125 (3.1%)	<0.001
Nicotine dependence	173,182 (25.6%)	16,900 (8.2%)	190,082 (21.5%)	<0.001
Deficiency anemia	126,933 (19.8%)	47,707 (24.3%)	174,640 (20.8%)	<0.001
Hypothyroidism	37,636 (5.9%)	23,261 (11.9%)	60,897 (7.3%)	<0.001
Previous PCI	65,403 (9.7%)	16,246 (7.8%)	81,649 (9.2%)	<0.001
Previous CABG	54,121 (8.0%)	19,955 (9.6%)	74,076 (8.4%)	<0.001
Previous MI	66,802 (9.9%)	20,568 (9.9%)	87,370 (9.9%)	<0.001
Cerebrovascular disease	68,736 (10.2%)	23,918 (11.5%)	92,654 (10.5%)	<0.001
Type of myocardial infarction				
STEMI	378,998 (56.1%)	97,889 (47.2%)	476,887 (54.0%)	<0.001
NSTEMI	296,494 (43.9%)	109,444 (52.8%)	405,938 (46.0%)	<0.001

Values are n (%) unless otherwise indicated.

Abbreviations as in Table 1.

a n < 11 data are not reported (NR) according to HCUP recommendations. In some cases, additional suppression is applied to prevent deductive disclosure.

The proportion of female patients decreased over the study period (54.3% to 45.3%; *P* < 0.001). Although the proportion of White patients decreased over the study period (84.6% to 76.6%), the proportions of Black, Hispanic, Asian or Pacific Islander, and Native American/Other patients increased (*P* < 0.001). The proportion of hospitalizations in rural and urban nonteaching hospitals decreased, which coincided with an increase in hospitalizations in urban teaching hospitals (42.2% to 77.3%; *P* < 0.001). The prevalence of most comorbidities increased over the study duration. ST-elevation myocardial infarction (STEMI) accounted for 47.2% of hospitalizations over the entire study period.

#### Outcomes

**Figure 2:**
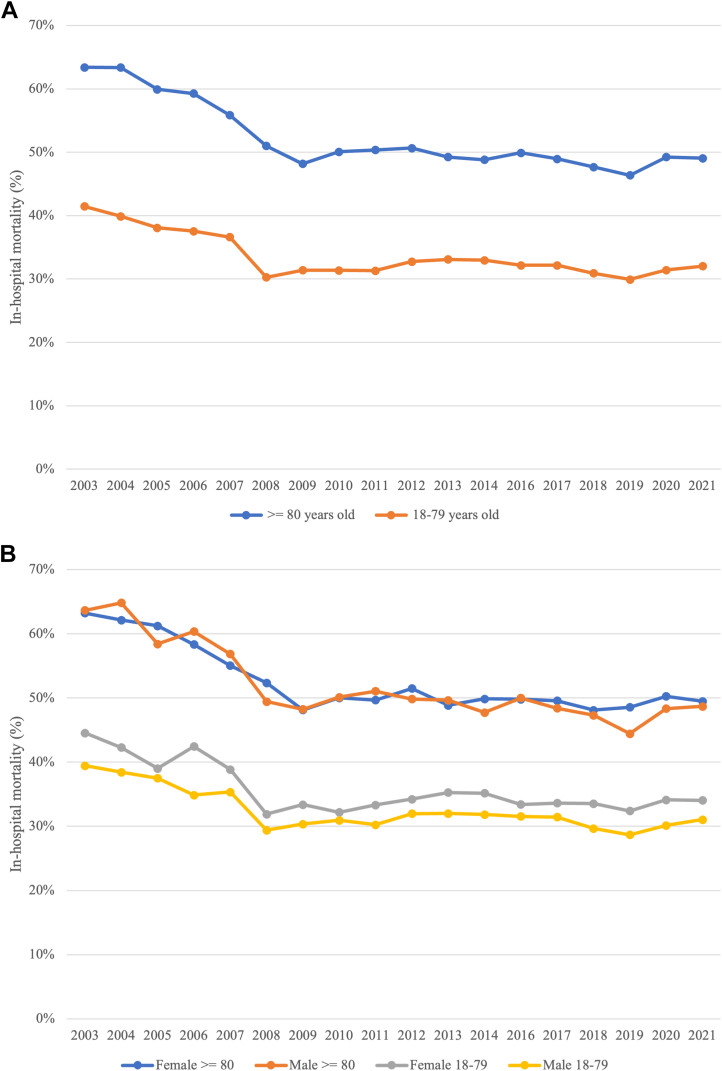
**Cardiogenic Shock With Acute Myocardial Infarction Mortality** Comparison of (A) overall in-hospital mortality and (B) sex-stratified in-hospital mortality in hospitalizations with cardiogenic shock and acute myocardial infarction in adults ≥80 years and 18 to 79 years in the United States from 2003 to 2021.

**Figure 3:**
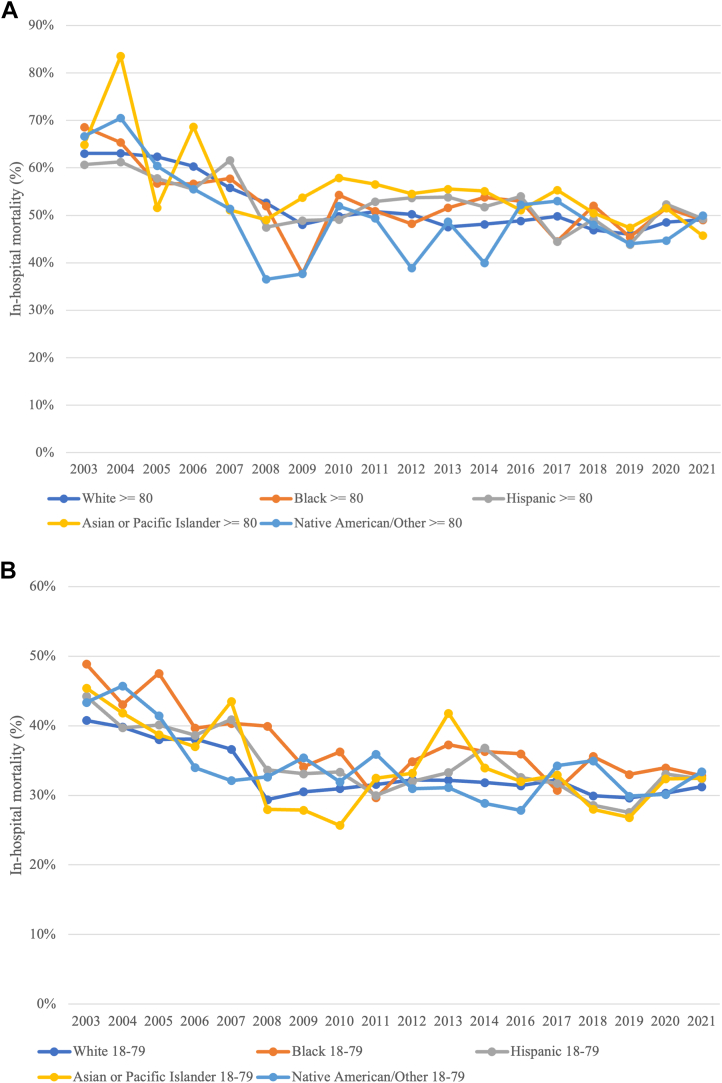
**Cardiogenic Shock With Acute Myocardial Infarction Racial/Ethnic Mortality** Comparison of in-hospital mortality in hospitalizations with cardiogenic shock and acute myocardial infarction in adults (A) ≥80 years and (B) 18 to 79 years in the United States from 2003 to 2021 stratified by race and ethnicity.

The in-hospital mortality for older patients decreased over the study period (61.4% to 49.0%; *P* < 0.001) with an overall in-hospital mortality of 51.8%. Over the study period, there were also significant increases in the utilization of invasive therapeutic interventions for older patients hospitalized with CS and AMI, including PCI (19.2% to 34.1%; *P* < 0.001) and pLVAD (0.2% to 8.3%), alongside a reduction in IABP usage (22.0% to 16.9%; *P* < 0.001). The utilization of ECMO also increased (0.1% to 0.3%). The proportion of associated complications increased with some more notable ones; including acute kidney injury (36.4% to 66.6%; *P* < 0.001), acute posthemorrhagic anemia (3.9% to 15.1%; *P* < 0.001), respiratory failure (40.9% to 65.2%; *P* < 0.001), and renal replacement therapy (4.0% to 8.2%; *P* = 0.014). Encounters for palliative care (2.0% to 32.4%; *P* < 0.001) and DNR orders (0.9% to 49.2%) also rose. In addition, the inflation-adjusted total hospitalization cost increased to more than twice the initial amount ($55,829 to $122,004; *P* < 0.001). The most common discharge locations for older patients were facilities (skilled nursing facilities, intermediate care facilities, or other) (27.0%) (Table 3, Table 4, Figure 2, Figure 3). Patients with a STEMI had greater mortality compared to those with non-ST-elevation myocardial infarction (NSTEMI) (57.3% vs 46.9%, respectively, *P* < 0.001) (Table 5).

**Table 3:** Outcomes of Hospitalizations With Cardiogenic Shock and Acute Myocardial Infarction of Patients Aged ≥80 Years in the United States, 2003-2021

	2003-2006	2007-2010	2011-2014	2016-2019	2020-2021	Total	*P* Value
Sample size	39,220 (18.9%)	42,539 (20.5%)	49,248 (23.8%)	51,640 (24.9%)	24,685 (11.9%)	207,333	
Cardiac arrest	4,662 (11.9%)	5,590 (13.1%)	8,795 (17.9%)	4,395 (8.5%)	3,485 (14.1%)	26,928 (13.0%)	<0.001
Cardiac tamponade	NR^a^	231 (0.5%)	316 (0.6%)	510 (1.0%)	310 (1.3%)	NR^a^	-
Pericarditis	169 (0.4%)	118 (0.3%)	94 (0.2%)	105 (0.2%)	NR^a^	NR^a^	-
Acute kidney injury	14,265 (36.4%)	20,452 (48.1%)	28,328 (57.5%)	32,145 (62.3%)	16,445 (66.6%)	111,635 (53.8%)	<0.001
Acute posthemorrhagic anemia	1,538 (3.9%)	2,743 (6.5%)	5,578 (11.3%)	7,670 (14.9%)	3,730 (15.1%)	21,258 (10.3%)	<0.001
Invasive mechanical ventilation	18,024 (46.0%)	19,073 (44.8%)	22,913 (46.5%)	22,235 (43.1%)	10,485 (42.5%)	92,730 (44.7%)	<0.001
Mechanical circulatory support	NR^a^	NR^a^	11,256 (22.9%)	12,040 (23.3%)	5,930 (24.0%)	NR^a^	-
Percutaneous left ventricular assist devices	NR^a^	81 (0.2%)	766 (1.6%)	3,295 (6.4%)	2,050 (8.3%)	NR^a^	-
Extracorporeal membrane oxygenation	NR^a^	NR^a^	59 (0.1%)	220 (0.4%)	70 (0.3%)	NR^a^	-
Intra-aortic balloon pump	8,619 (22.0%)	9,814 (23.1%)	10,671 (21.7%)	9,200 (17.8%)	4,165 (16.9%)	42,469 (20.5%)	<0.001
Renal replacement therapy	1,567 (4.0%)	2,229 (5.2%)	2,892 (5.9%)	3,750 (7.3%)	2,020 (8.2%)	12,458 (6.0%)	0.014
Encounter for palliative care	765 (2.0%)	3,429 (8.1%)	9,581 (19.5%)	14,850 (28.8%)	7,995 (32.4%)	36,620 (17.7%)	<0.001
Do not resuscitate	NR^a^	368 (0.9%)	14,421 (29.3%)	22,990 (44.5%)	12,155 (49.2%)	49,935 (24.1%)	-
Percutaneous coronary intervention	7,531 (19.2%)	11,465 (27.0%)	14,731 (29.9%)	17,440 (33.8%)	8,425 (34.1%)	59,592 (28.7%)	<0.001
Coronary artery bypass graft	2,784 (7.1%)	3,108 (7.3%)	3,647 (7.4%)	3,895 (7.5%)	1,930 (7.8%)	15,364 (7.4%)	<0.001
Gastrostomy	903 (2.3%)	970 (2.3%)	1,055 (2.1%)	1,430 (2.8%)	705 (2.9%)	5,062 (2.4%)	0.354
Tracheostomy	1,075 (2.7%)	1,127 (2.7%)	1,022 (2.1%)	665 (1.3%)	280 (1.1%)	4,169 (2.0%)	<0.001
GI bleeding	3,121 (8.0%)	3,485 (8.2%)	3,411 (6.9%)	3,685 (7.1%)	1,680 (6.8%)	15,382 (7.4%)	0.004
Pulmonary embolism	425 (1.1%)	439 (1.0%)	769 (1.6%)	840 (1.6%)	435 (1.8%)	2,907 (1.4%)	0.011
Respiratory failure	16,042 (40.9%)	19,895 (46.8%)	28,762 (58.4%)	32,375 (62.7%)	16,095 (65.2%)	113,169 (54.6%)	<0.001
In-hospital mortality	24,045 (61.4%)	21,790 (51.2%)	24,459 (49.7%)	24,875 (48.2%)	12,105 (49.0%)	107,274 (51.8%)	<0.001
Length of stay	4.6 (1.1-10.3)	5.2 (1.4-10.8)	4.8 (1.4-10.2)	4.6 (1.4-9.7)	4.5 (1.4-9.6)	4.7 (1.3-10.1)	<0.001
Total hospitalization cost (USD 2021)	55,829 (23,069-123,965)	74,842 (32,089-152,983)	88,536 (38,902-176,969)	109,386 (50,840-220,500)	122,004 (55,059-244,186)	86,801 (37,140-180,119)	<0.001
Discharge disposition							<0.001
Routine transfer to home or self-care	2,663 (6.8%)	3,141 (7.4%)	3,456 (7.0%)	3,795 (7.4%)	2,050 (8.3%)	15,104 (7.3%)	
Transfer to short-term hospital	1,484 (3.8%)	1,659 (3.9%)	1,974 (4.0%)	2,440 (4.7%)	960 (3.9%)	8,517 (4.1%)	
Transfer to facility (skilled nursing facility, intermediate care facility, etc)	8,343 (21.3%)	12,110 (28.5%)	14,852 (30.2%)	14,805 (28.7%)	5,910 (23.9%)	56,020 (27.0%)	
Died	24,045 (61.4%)	21,790 (51.2%)	24,459 (49.7%)	24,875 (48.2%)	12,105 (49.0%)	107,274 (51.8%)	
Home health care/other	2,612 (6.7%)	3,822 (9.0%)	4,476 (9.1%)	5,680 (11.0%)	3,660 (14.8%)	20,249 (9.8%)	

Values are n (%) unless otherwise indicated.

Variables included in the multivariable regression model: time period, sex, race/ethnicity, insurance type, zip code income quartile, hospital location, region of hospital, congestive heart failure, valvular disease, chronic pulmonary disease, obesity, dementia, dyslipidemia, diabetes mellitus, hypertension, liver disease, peripheral vascular disease, renal failure, malnutrition, atrial fibrillation, history of malignancy, coagulopathy, solid tumor without metastasis, alcohol abuse, nicotine dependence, deficiency anemia, hypothyroidism, previous PCI, previous CABG, previous MI, cerebrovascular disease, STEMI.

*P* values for all outcomes are derived from multivariable regression analysis.

a n < 11 data are not reported (NR) according to HCUP recommendations. In some cases, additional suppression is applied to prevent deductive disclosure.

**Table 4:** Outcomes of Hospitalizations With Cardiogenic Shock and Acute Myocardial Infarction of Patients Aged 18-79 vs ≥ 80 Years in the United States, 2003-2021

	Age 18-79 Years	Age ≥80 Years	Total	Unadjusted OR (95% CI)	*P* Value	Adjusted OR (95% CI)	*P* Value
Sample size	675,491 (76.5%)	207,333 (23.5%)					
Cardiac arrest	119,339 (17.7%)	26,928 (13.0%)	146,267 (16.6%)	0.69 (0.67-0.72)	<0.001	0.79 (0.76-0.82)	<0.001
Cardiac tamponade	6,875 (1.0%)	NR^a^	NR^a^	-	-	-	-
Pericarditis	2,618 (0.4%)	NR^a^	NR^a^	-	-	-	-
Acute kidney injury	317,058 (46.9%)	111,635 (53.8%)	428,694 (48.6%)	1.32 (1.29-1.35)	<0.001	1.22 (1.18-1.25)	<0.001
Acute posthemorrhagic anemia	103,392 (15.3%)	21,258 (10.3%)	124,651 (14.1%)	0.63 (0.61-0.66)	<0.001	0.65 (0.62-0.68)	<0.001
Invasive mechanical ventilation	359,223 (53.2%)	92,730 (44.7%)	451,952 (51.2%)	0.71 (0.70-0.73)	<0.001	0.72 (0.70-0.74)	<0.001
Mechanical circulatory support	280,061 (41.5%)	NR^a^	NR^a^	-	-	-	-
Percutaneous left ventricular assist devices	45,849 (6.8%)	NR^a^	NR^a^	-	-	-	-
Extracorporeal membrane oxygenation	13,476 (2.0%)	NR^a^	NR^a^	-	-	-	-
Intra-aortic balloon pump	237,513 (35.2%)	42,469 (20.5%)	279,981 (31.7%)	0.48 (0.46-0.49)	<0.001	0.60 (0.58-0.62)	<0.001
Renal replacement therapy	64,473 (9.5%)	12,458 (6.0%)	76,931 (8.7%)	0.61 (0.58-0.63)	<0.001	0.46 (0.44-0.49)	<0.001
Encounter for palliative care	61,468 (9.1%)	36,620 (17.7%)	98,088 (11.1%)	2.14 (2.07-2.22)	<0.001	1.75 (1.68-1.83)	<0.001
Do not resuscitate	73,939 (11.0%)	49,935 (24.1%)	123,873 (14.0%)	2.58 (2.50-2.66)	<0.001	2.13 (2.05-2.21)	<0.001
Percutaneous coronary intervention	289,712 (42.9%)	59,592 (28.7%)	349,304 (39.6%)	0.54 (0.52-0.55)	<0.001	0.69 (0.66-0.71)	<0.001
Coronary artery bypass graft	119,168 (17.6%)	15,364 (7.4%)	134,532 (15.2%)	0.37 (0.36-0.39)	<0.001	0.44 (0.42-0.46)	<0.001
Gastrostomy	20,146 (3.0%)	5,062 (2.4%)	25,208 (2.9%)	0.81 (0.76-0.87)	<0.001	0.79 (0.72-0.86)	<0.001
Tracheostomy	27,267 (4.0%)	4,169 (2.0%)	31,436 (3.6%)	0.49 (0.45-0.53)	<0.001	0.52 (0.48-0.57)	<0.001
GI bleeding	46,587 (6.9%)	15,382 (7.4%)	61,969 (7.0%)	1.08 (1.04-1.13)	<0.001	1.04 (0.98-1.09)	0.179
Pulmonary embolism	12,297 (1.8%)	2,907 (1.4%)	15,204 (1.7%)	0.77 (0.70-0.84)	<0.001	0.80 (0.72-0.89)	<0.001
Respiratory failure	404,779 (59.9%)	113,169 (54.6%)	517,948 (58.7%)	0.80 (0.79-0.82)	<0.001	0.79 (0.76-0.81)	<0.001
In-hospital mortality	222,727 (33.0%)	107,274 (51.8%)	330,001 (37.4%)	2.18 (2.13-2.23)	<0.001	1.89 (1.83-1.94)	<0.001
Length of hospital stay (days)	6.8 (2.5-13.2)	4.7 (1.3-10.1)	6.3 (2.2-12.5)		<0.001		<0.001
Total hospitalization cost (USD 2021)	153,138 (79,627-286,847)	86,801 (37,140-180,119)	136,051 (67,052-261,850)		<0.001		<0.001
Discharge disposition							
Routine transfer to home or self-care	183,333 (27.2%)	15,104 (7.3%)	198,438 (22.5%)	0.21 (0.20-0.22)	<0.001	0.37 (0.35-0.39)	<0.001
Transfer to short-term hospital	54,752 (8.11%)	8,517 (4.1%)	63,270 (7.2%)	0.49 (0.46-0.51)	<0.001	0.49 (0.46-0.52)	<0.001
Transfer to facility (skilled nursing facility, intermediate care facility, etc)	128,421 (19.0%)	56,020 (27.0%)	184,441 (20.9%)	1.58 (1.54-1.62)	<0.001	1.13 (1.10-1.17)	<0.001
Died	222,727 (33.0%)	107,274 (51.8%)	330,001 (37.4%)	2.18 (2.13-2.23)	<0.001	1.89 (1.83-1.94)	<0.001
Home health care/other	85,700 (12.7%)	20,249 (9.8%)	105,950 (12.0%)	0.75 (0.72-0.77)	<0.001	0.74 (0.70-0.77)	<0.001

Hospitalizations of patients aged 18 to 79 were used as the reference group for calculation of ORs. ORs were not calculated for numeric variables (length of hospital stay and total hospitalization cost).

Values are n (%) unless otherwise indicated. Variables included in the multivariable regression model: time period, sex, race/ethnicity, insurance type, zip code income quartile, hospital location, region of hospital, congestive heart failure, valvular disease, chronic pulmonary disease, obesity, dementia, dyslipidemia, diabetes mellitus, hypertension, liver disease, peripheral vascular disease, renal failure, malnutrition, atrial fibrillation, history of malignancy, coagulopathy, solid tumor without metastasis, alcohol abuse, nicotine dependence, deficiency anemia, hypothyroidism, previous PCI, previous CABG, previous MI, cerebrovascular disease, STEMI.

a n < 11 data are not reported (NR) according to HCUP recommendations. In some cases, additional suppression is applied to prevent deductive disclosure.

**Table 5:** Outcomes of Hospitalizations With Cardiogenic Shock and Acute Myocardial Infarction of Patients Aged ≥80 Years Old With STEMI Vs NSTEMI, 2003-2021

	STEMI	NSTEMI	*P* Value
Sample size	97,889 (47.2%)	109,444 (52.8%)	
Cardiac arrest	14,915 (15.2%)	12,012 (11.0%)	<0.001
Cardiac tamponade	650 (0.7%)	715 (0.7%)	0.938
Pericarditis	312 (0.3%)	219 (0.2%)	0.076
Acute kidney injury	44,524 (45.5%)	67,111 (61.3%)	<0.001
Acute posthemorrhagic anemia	7,861 (8.0%)	13,397 (12.2%)	<0.001
Invasive mechanical ventilation	43,499 (44.4%)	49,231 (45.0%)	0.630
Mechanical circulatory support	28,897 (29.5%)	18,813 (17.2%)	<0.001
Percutaneous left ventricular assist devices	2,789 (2.9%)	3,418 (3.1%)	0.002
Extracorporeal membrane oxygenation	150 (0.2%)	200 (0.2%)	0.224
Intra-aortic balloon pump	26,542 (27.1%)	15,927 (14.6%)	<0.001
Renal replacement therapy	3,756 (3.8%)	8,701 (8.0%)	<0.001
Encounter for palliative care	15,594 (15.9%)	21,026 (19.2%)	0.003
Do not resuscitate	20,170 (20.6%)	29,765 (27.2%)	<0.001
Percutaneous coronary intervention	41,094 (42.0%)	18,498 (16.9%)	<0.001
Coronary artery bypass graft	5,733 (5.9%)	9,631 (8.8%)	<0.001
Gastrostomy	1,865 (1.9%)	3,197 (2.9%)	0.031
Tracheostomy	1,633 (1.7%)	2,536 (2.3%)	0.211
GI bleeding	7,237 (7.4%)	8,145 (7.4%)	0.467
Pulmonary embolism	1,111 (1.1%)	1,796 (1.6%)	<0.001
Respiratory failure	47,186 (48.2%)	65,983 (60.3%)	<0.001
In-hospital mortality	55,995 (57.3%)	51,279 (46.9%)	<0.001
Length of stay in days	3.4 (0.7-8.4)	6.0 (2.2-11.5)	<0.001
Total hospitalization cost (USD 2021)	81,652 (32.540-165,714)	91,635 (40,808-195,207)	<0.001
Discharge disposition			<0.001
Routine transfer to home or self-care	8,229 (8.4%)	6,875 (6.3%)	
Transfer to short-term hospital	3,442 (3.5%)	5,075 (4.6%)	
Transfer to facility (skilled nursing facility, intermediate care facility, etc)	21,881 (22.4%)	34,138 (31.2%)	
Died	55,995 (57.3%)	51,279 (46.9%)	
Home health care/other	8,226 (8.4%)	12,024 (11.0%)	

Values are n (%) unless otherwise indicated. Variables included in the multivariable regression model: time period, sex, race/ethnicity, insurance type, zip code income quartile, hospital location, region of hospital, congestive heart failure, valvular disease, chronic pulmonary disease, obesity, dementia, dyslipidemia, diabetes mellitus, hypertension, liver disease, peripheral vascular disease, renal failure, malnutrition, atrial fibrillation, history of malignancy, coagulopathy, solid tumor without metastasis, alcohol abuse, nicotine dependence, deficiency anemia, hypothyroidism, previous PCI, previous CABG, previous MI, cerebrovascular disease, STEMI.

*P* values for all outcomes are derived from multivariable regression analysis.

^a^n < 11 data are not reported (NR) according to HCUP recommendations. In some cases, additional suppression is applied to prevent deductive disclosure.

### Relatively younger patient hospitalizations

#### Clinical characteristics of hospitalizations

A total of 675,491 hospitalizations with CS and AMI between the ages of 18 to 79 years from 2003 to 2021 were identified. This age group accounted for 76.5% of all identified hospitalizations with CS and AMI. The median age was 65, 34.3% were female, and 72.2% were White. The majority were Medicare beneficiaries (53.6%) and were admitted to urban teaching hospitals (65.9%). The Southern region was the most common location for these hospitalizations (40.3%) (Supplemental Table 3).

### Outcomes

The in-hospital mortality decreased over the study period (39.2% to 31.7%; *P* < 0.001) with an overall in-hospital mortality of 33.0%. There were concomitant increases in acute kidney injury (29.7% to 57.5%; *P* < 0.001), acute posthemorrhagic anemia (4.7% to 23.0%; *P* < 0.001), pLVAD use (0.1% to 14.6%; *P* < 0.001), ECMO utilization (0.2% to 3.3%; *P* < 0.001), renal replacement therapy (6.0% to 12.2%; *P* < 0.001), palliative care encounters (0.7% to 15.8%; *P* < 0.001), DNR orders (0.3% to 20.8%), PCI (36.9% to 41.6%; *P* < 0.001), and respiratory failure (44.0% to 66.2%; *P* < 0.001) alongside a decline in IABP usage (44.1% to 24.7%; *P* < 0.001). Total MCS usage, comprising pLVAD, ECMO, and IABP, decreased (44.1% to 38.2%; *P* < 0.001). The inflation-adjusted total hospitalization cost increased ($108,258 to $196,338; *P* < 0.001), and the most common discharge location was routine transfer to home or self-care (27.2%) (Supplemental Table 4). Relatively younger patients aged 18 to 79 had higher mortality from STEMI compared to that from an NSTEMI (34.0% vs 31.7%; *P* < 0.001) (Supplemental Table 5).

### Comparison of older and relatively younger patient hospitalizations

During the entire study period, when compared to relatively younger patients, older patients ≥80 years had a greater incidence of in-hospital mortality (adjusted OR [aOR]: 1.89; 95% CI: 1.83-1.94), acute kidney injury (aOR: 1.22; 95% CI: 1.18-1.25), DNR orders (aOR: 2.13; 95% CI: 2.05-2.21), and palliative care encounters (aOR: 1.75; 95% CI: 1.68-1.83). Older patients had lower utilization of PCI (aOR: 0.69; 95% CI: 0.66-0.71), CABG (aOR: 0.44; 95% CI: 0.42-0.46), and had lower total inflation-adjusted hospitalization cost ($86,801 vs $153,138; *P* < 0.001).

## Discussion

The present study is unique compared to prior national-level studies on CS as it focused on the subgroup of CS with AMI in older patients ≥80 years of age. This study reports several key findings. Between 2003 and 2021, the number of hospitalizations with CS and AMI in older patients increased significantly, although older adults constituted a decreasing percentage of all adult hospitalizations for CS and AMI over time. This increase in CS in older adults was sustained when the percentage of CS among all AMI hospitalizations was analyzed.

Among older adults, there was a decrease in in-hospital mortality over time, as well as an increase in the utilization of PCI, CABG, pLVAD, and ECMO. Complications including acute kidney injury, acute posthemorrhagic anemia, and renal replacement therapy increased. Concurrently, there were prominent increases in the utilization of palliative care and DNR orders. Compared to those 18 to 79 years of age, older adults had lower utilization of revascularization and MCS, greater utilization of palliative care and DNR orders, and greater in-hospital mortality. These results demonstrate important findings in the care utilization and outcomes for older individuals hospitalized with CS and AMI.

Our finding of the increase in CS with AMI is consistent with other studies showing a significant increase in hospitalizations attributed to CS.^1^ Similar increases in CS incidence have been observed in subgroups based on STEMI and NSTEMI cases from 2000 to 2017.^10^^,^^11^ This rise in CS in AMI hospitalizations is hypothesized to be due to increased awareness about timely diagnosis and the implementation of shock teams in specialized centers and algorithms, leading to the identification of more CS patients.^12^^,^^13^ Increased complexity and comorbidities in the patient population and changes in demographics, including an aging population and increased survival rates from initial cardiac events, are also thought to contribute to increasing CS hospitalizations.^14^^,^^15^ Additionally, the increase in CS with AMI rates may be partly attributed to changes in coding practices, which are likely influenced by reimbursement.

The declining in-hospital mortality in this study is consistent with some prior studies of hospitalizations with CS with and without AMI.^1^ Early revascularization due to the increased availability of cardiac catheterization facilities and advancements in revascularization techniques, such as the increased use of coronary angiography and PCI, may primarily have driven a decline, that is, the use of PCI in STEMI complicated with CS increased from 38.6% to 70.6% between 2000 and 2017.^11^^,^^16^ Similarly, in NSTEMI-CS, PCI use increased from 14.8% to 31.6%.^10^

However, recent data from worldwide observational studies demonstrate varying incidence and mortality for CS from AMI, ranging from 3% to 15% of all AMI cases.^17^ Data from 3 nationwide French registries demonstrate a decrease in the prevalence of CS complicating AMI from 5.9% in 2005 to 2.8% in 2015, with no change in in-hospital mortality despite increased use of invasive strategies.^18^ Furthermore, results from an analysis of the Cath-PCI registry investigating patients with CS from AMI undergoing PCI from 2005 to 2013 showed increased in-hospital mortality from 27.6% to 30.6% over the study period.^19^ It is also noteworthy that after adjusting for out-of-hospital cardiac arrest, CS from AMI mortality has been shown to be decreasing.^20^ Differences in the results of the present study and those from prior research may be attributable to factors including the focus of this study on a subpopulation of patients ≥80 years of age, differences in the location of aforementioned studies, or variability in the coding or defining of CS.

Revascularization rates in this study are similar to those reported from prior cohorts and mirror the rates seen in other high-risk conditions such as AMI in the presence of coexisting heart failure.^21^ In addition, the use of temporary MCS devices for CS due to AMI has gained traction over the past 2 decades. With previously unclear benefits, the recent finding of decreasing mortality from microaxial flow pumps in the DanGer Shock trial is another potential reason for improvement seen in CS mortality since these devices were introduced in the initial part of the present study timeline.^1^^,^^22^ The observed increase in associated complications such as acute kidney injury, acute posthemorrhagic anemia, and renal replacement therapy may be secondary to the demonstrated rise in PCI and MCS application.^22^ Increased awareness, advances in critical care management, and the introduction of more robust percutaneous MCS platforms, along with implementing CS treatment algorithms and collaborative efforts to improve prehospital and in-hospital care, may have played a role in reducing CS mortality.^23^^,^^24^

According to the present analysis, the inflation-adjusted total hospitalization cost for hospitalizations with CS and AMI has significantly increased in recent years. This increase has been observed elsewhere, such as in the United States, where the average hospital cost for patients with CS complicating STEMI rose from $35,892 in 2003 to $45,625 in 2010.^25^ In Ontario, Canada, the median inpatient cost per patient with AMI-related CS was $23,912, with a median total 1-year cost of $37,913.^26^ This is likely driven by factors including increased use of advanced therapies and interventions (PCI, MCS such as ECMO/pLVAD), higher complexity of cases, and prolonged utilization of critical care facilities.^11^^,^^25^^,^^27^

Utilization of palliative care and DNR orders has increased considerably during the study period, more so in older patients than in relatively younger patients. Similar to the findings in the present study, an analysis of palliative care utilization in CS complicating AMI within the United States from 2000 to 2014 also demonstrated a rise in palliative care use despite a limited overall usage.^28^ This increase is intertwined with the American College of Cardiology, American Heart Association, and Heart Failure Society of America's recommendation to involve multidisciplinary teams, including palliative care specialists, in managing CS to ensure holistic patient care and discussions about prognosis and patient preferences.^29^ Although increasing DNR orders and engagement with palliative care services may represent a growing emphasis on goal-concordant patient care and greater availability of palliative care services, it is possible that factors including temporal variation in coding practices and improved capture of palliative care consultation and DNR orders also contributed to this observed finding.^30^ Palliative care involvement in CS is associated with reduced utilization of invasive procedures, lower hospitalization costs, and lower 30-day readmission rates.^31^^,^^32^

These findings may have implications for the care of the growing number of older adults hospitalized with CS and AMI. In light of the aging population in the United States and the growing number of comorbidities among older adults hospitalized with CS and AMI, additional efforts may be of value to optimize comorbidities in this patient population. Appropriately selected older patients continue to derive benefits from primary and secondary prevention strategies, such as optimization of hypertension^33^ and utilization of statin therapy,^34^ suggesting that older age should not be a contraindication to risk factor optimization. We demonstrate that complex interventions including revascularization and MCS may be appropriate and are increasingly utilized in older adults with CS and AMI. Furthermore, decreasing in-hospital mortality rates in this age group were demonstrated in this analysis, which suggests that age alone should not preclude complex care consideration among carefully selected older patients. However, there are limited data on the benefits of complex interventions in older adults, including revascularization in the setting of shock and invasive management in the setting of non–ST-segment elevation AMI.^16^^,^^35^ Therefore, future studies focusing on the treatment approaches to CS in the growing population of older adults would be of value. Additionally, the current results may be beneficial in highlighting the high mortality among older adults hospitalized with CS and AMI, which can assist in prognostication and facilitate an increased focus on goals of care discussion.

## Study Limitations

Although the National Inpatient Sample database effectively evaluates demographics, diagnoses, and outcomes, several limitations to this study related to the National Inpatient Sample database should be acknowledged.^36^ First, ICD-9 and ICD-10 coding errors and misclassifications can result in erroneous data and results. Second, increases in coding secondary to factors including financial and institutional incentives across years may have impacted the results. Third, patients with CS and AMI may not necessarily have CS due to AMI. Fourth, this study includes the transition from ICD-9 to ICD-10 codes in 2015, which may have resulted in variations in coding and classifications. Fifth, prior to October 2005, ICD-9 codes for AMI did not differentiate between STEMI and NSTEMI, resulting in an inability to subclassify AMI during this time. Sixth, although type 2 AMI was excluded from the analysis, the ICD-10 codes to identify type 2 AMI were first introduced in October 2017, which likely resulted in the erroneous inclusion of these patients before this period. Seventh, the National Inpatient Sample database does not provide access to specified granular data on many patient parameters, such as lab values, imaging results, or pharmacotherapy regimens. Eighth, the severity of CS could not be assessed, such as with the Society Cardiovascular Angiography Interventions shock scale, which may confound results. Ninth, the unit of analysis with the National Inpatient Sample database is hospitalizations rather than unique patients which results in the inability to investigate on a per-patient basis. Tenth, the grouping of all patients 18 to 79 years of age into a single category may conceal important age-related heterogeneity within this large age range. Eleventh, due to the large sample size resulting in the possibility of small group differences reaching statistical significance, it is important for the *P* values to be interpreted with caution within the context of effect size and clinical relevance. Lastly, the National Inpatient Sample database does not provide data after hospital discharge, which may provide a useful outlook on long-term outcomes.

## Conclusions

Hospitalizations with CS and AMI in older individuals have increased from 2003 to 2021 and older patients continue to have a higher hospitalization burden than relatively younger patients. In-hospital mortality rates for older individuals have reduced over time. Increases in revascularization with PCI rates were observed with a concomitant increase in complications. Furthermore, there was a significantly increasing emphasis on palliative care involvement in older patients. These results may have important implications for the growing number of older adults hospitalized with CS and AMI.

Perspectives **COMPETENCY IN SYSTEMS-BASED PRACTICE:** CS is most commonly secondary to AMI and is associated with high mortality and morbidity. Although older patients represent an important demographic of patients with CS from an AMI, there are little data on their outcomes as they have been largely excluded from clinical trials.

**TRANSLATIONAL OUTLOOK:** Older patients’ hospitalizations with CS and AMI increased from 2003 to 2021. There was an increase in the utilization of revascularization and invasive therapeutic interventions such as PCI and MCS over the same time period in older patients.

## Funding support and author disclosures

The authors have reported that they have no relationships relevant to the contents of this paper to disclose.
