# Supplementary material for: Cardiogenic Shock With Acute Myocardial Infarction Among Older Adults in the United States
Source: JACC Adv. 2025 Aug 19;4(9):102078. doi: 10.1016/j.jacadv.2025.102078 (PMC12395060; doi:10.1016/j.jacadv.2025.102078)
Supplement: Supplementary Material [file mmc1.docx]

**Supplemental Table 1**. ICD-9/PCS codes used in the analysis.

| **Variables** | **ICD-9/PCS Codes** |
| --- | --- |
| Acute myocardial infarction | 410.00, 410.01, 410.10, 410.11, 410.20, 410.21, 410.30, 410.31, 410.40, 410.41, 410.50, 410.51, 410.60, 410.61, 410.80, 410.81, 410.90, 410.91, 410.70, 410.71 |
| Cardiogenic shock | 785.51, 998.01 |
| Congestive heart failure | 428.1, 428.20, 428.0, 428.21, 428.22, 428.23, 428.30, 428.31, 428.32, 428.33, 428.40, 428.41, 428.42, 428.43, 428.9, 402.11, 402.01, 398.91,  402.91, 404.01, 404.03, 404.11, 404.13, 404.91, 404.93 |
| Valvular disease | Elixhauser comorbidity |
| Chronic pulmonary disease | Elixhauser comorbidity |
| Obesity | Elixhauser comorbidity |
| Dementia | 290, 290.1, 290.11, 290.12, 290.13, 290.2, 290.21, 290.3, 290.4, 290.41, 290.42, 290.43, 291.2, 292.82, 294.1, 294.11, 294.2, 294.21, 331.82, 331.0, 331.11, 331.6, 331.7, 331.81, 331.83  331.89, 331.9, 046.0, 046.11, 046.19, 046.2, 046.3, 331.19, 333.0 |
| Dyslipidemia | 272.1, 272.2, 272.3, 272.4, 272.5, 272.9, 272.0, 272.8 |
| Iron deficiency | 280.0, 280.8, 280.1, 280.9 |
| Diabetes Mellitus | Elixhauser comorbidity |
| Hypertension | Elixhauser comorbidity |
| Liver disease | Elixhauser comorbidity |
| Neurological disorders | Elixhauser comorbidity |
| Peripheral vascular disease | Elixhauser comorbidity |
| Renal failure | Elixhauser comorbidity |
| Malnutrition | 262, 263.0, 263.1, 263.9, 263.8 |
| Atrial fibrillation | 427.31 |
| History of malignancy | V10.00, V10.03, V10.91, V10.04, V10.05, V10.06, V10.07, V10.09, V10.12, V10.11, V10.20, V10.21, V10.22, V10.91, V10.29, V10.3, V10.40, V10.41, V10.42, V10.43, V10.44, V10.45, V10.46, V10.47, V10.48, V10.49, V10.50, V10.51, V10.53, V10.59, V10.91, V10.52, V10.59, V10.60, V10.72, V10.79, V10.01, V10.02, V10.91, V10.83, V10.81, V10.89, V10.84, V10.85, V10.86, V10.87, V10.88, V10.89, V10.90 |
| AIDS | Elixhauser comorbidity |
| Coagulopathy | Elixhauser comorbidity |
| Solid tumor without metastasis | Elixhauser comorbidity |
| Drug abuse | Elixhauser comorbidity |
| Alcohol abuse | Elixhauser comorbidity |
| Nicotine dependence | Elixhauser comorbidity |
| Deficiency anemia | Elixhauser comorbidity |
| Hypothyroidism | Elixhauser comorbidity |
| Previous PCI | V45.82 |
| Previous CABG | V45.81, 414.02, 414.03, 414.04, 414.05, 414.07 |
| Previous MI | 412, 410.02, 410.12, 410.22, 410.32, 410.42, 410.52, 410.62, 410.72, 410.82, 410.92 |
| Cerebrovascular disease | 430, 431, 432, 432.1, 432.9, 433.01, 433.11, 433.2, 433.21, 433.31, 433.81, 433.91, 434.01, 434.11, 434.91, 435, 435.1, 435.2, 435.3, 435.8, 436, 437.1, 438.00, 438.1, 438.11, 438.12, 438.13, 438.14, 438.19, 438.2, 438.21, 438.22, 438.3, 438.31, 438.32, 438.4, 438.41, 438.42, 438.5, 438.51, 438.52, 438.53, 438.6, 438.7, 438.81, 438.82, 438.83, 438.84, 438.85, 438.89, 438.9, 997.02, 852, 852.01, 852.02, 852.03, 852.04, 852.05, 852.06, 852.09, 852.1, 852.11, 852.12, 852.13, 852.14, 852.15, 852.16, 852.19, 852.2, 852.21, 852.22, 852.23, 852.24, 852.25, 852.26, 852.29, 852.3, 852.31, 852.32, 852.33, 852.34, 852.35, 852.36, 852.39, 852.4, 852.41, 852.42, 852.43, 852.44, 852.45, 852.46, 852.49, 852.5, 852.51, 852.52, 852.53, 852.54, 852.55, 852.56, 852.59, 853, 853.01, 853.02, 853.03, 853.04, 853.05, 853.06, 853.09, 853.1, 853.11, 853.12, 853.13, 853.14, 853.15, 853.16, 853.19, 854, 854.01, 854.02, 854.03, 854.04, 854.05, 854.06, 854.09, 854.1, 854.11, 854.12, 854.13, 854.14, 854.15, 854.16, 854.19, 342, 342.01, 342.02, 342.1, 342.11, 342.12, 342.8, 342.81, 342.82, 342.9, 342.91, 342.92, 344.3, 344.31, 344.32, 344.4, 344.41, 344.42, 344.5, 344.81, 334.3, 784.3, V12.54 |
| Cardiac arrest | 427.5 |
| Complete atrioventricular block | 426 |
| Cardiac tamponade | 423.3 |
| Pericarditis | 420.91, 420.90, 420.99 |
| Acute Kidney Injury/Failure | 584.5, 584.6, 584.7, 584.8, 584.9 |
| Acute Post Hemorrhagic Anemia | 285.1 |
| Invasive mechanical ventilation | 96.70, 96.71, 96.72, 96.04 |
| Percutaneous left ventricular assist devices | 37.68 |
| Extracorporeal Membrane Oxygenation | 39.65 |
| Intra-Aortic Balloon Pump | 37.61 |
| Durable Left Ventricular Assist Devices | 37.52, 37.66 |
| Renal Replacement Therapy | 39.95 |
| Encounter for Palliative Care | V66.7 |
| Do Not Resuscitate | V49.86 |
| Percutaneous Coronary Intervention | 00.66, 36.07, 36.06, 17.55 |
| Coronary Artery Bypass Graft | 36.10, 36.11, 36.12, 36.13, 36.14, 36.15, 36.16, 36.17, 36.19 |
| Gastrostomy | 43.1, 43.11, 43.19, 44.32 |
| Tracheostomy | 31.1, 31.21, 31.29 |
| GI Bleeding | 456.0, 456.20, 531.00, 531.20, 531.40, 531.60, 532.00, 532.20, 532.40, 532.60, 533.00, 533.20, 533.40, 533.60, 534.00, 534.20  534.40, 534.60, 535.01, 535.31, 535.11, 535.21, 535.41, 535.51  535.61, 569.3, 578.0, 578.1, 578.9 |
| Pulmonary embolism | 415.12, 415.13, 415.19, 415.1, 415.11 |
| Respiratory Failure | 518.51, 518.52, 518.53, 518.81, 518.84, 518.82, 770.89 |

**Supplemental Table 2**: ICD-10/PCS codes used in the analysis

| **Variables** | **ICD-10/PCS Codes** |
| --- | --- |
| Acute myocardial infarction | I21.01, I21.02, I21.09, I21.11, I21.19, I21.21, I21.29, I21.3, I22.0, I22.1, I22.2, I22.8, I22.9, I21.4 |
| Cardiogenic shock | R57.0, T81.11XA |
| Congestive heart failure | I50.1, I50.2, I50.20, I50.21, I50.22, I50.23, I50.3, I50.30, I50.31  I50.32, I50.33, I50.4, I50.40, I50.41, I50.42, I50.43, I50.8, I50.81  I50.810, I50.811, I50.812, I50.813, I50.814, I50.82, I50.83, I50.84  I50.89, I50.9, I09.81, I11.0, I13.0, I13.2 |
| Valvular disease | Elixhauser comorbidity |
| Chronic pulmonary disease | Elixhauser comorbidity |
| Obesity | Elixhauser comorbidity |
| Dementia | F01.50, F01.51, F02.80, F02.81, F03.90, F03.91, F10.27, F13.27  F13.97, F18.17, F18.27, F18.97, F19.17, F19.27, F19.97, G30, G30.0, G30.1, G30.8, G30.9, G31 , G31.01, G31.09, G31.2, G31.83, G31.1, G31.2, G31.81, G31.82, G31.85, G31.89, G31.9  A81.00, A81.01, A81.09, A81.1, A81.2 |
| Dyslipidemia | E78, E78.0, E78.00, E78.01, E78.1, E78.2, E78.3, E78.4, E78.41  E78.49, E78.5, E78.6, E78.7, E78.70, E78.71, E78.72, E78.79  E78.8, E78.81, E78.89, E78.9 |
| Iron deficiency | D50, D50.0, D50.1, D50.8, D50.9 |
| Diabetes Mellitus | Elixhauser comorbidity |
| Hypertension | Elixhauser comorbidity |
| Liver disease | Elixhauser comorbidity |
| Neurological disorders | Elixhauser comorbidity |
| Peripheral vascular disease | Elixhauser comorbidity |
| Renal failure | Elixhauser comorbidity |
| Malnutrition | E43, E44.0, E44.1, E46 |
| Atrial fibrillation | I48.0, I48.1, I48.2, I48.91, I48.11, I48.19, I48.20, I48.21 |
| History of malignancy | Z85, Z85.0, Z85.00, Z85.01, Z85.02, Z85.020, Z85.028, Z85.03  Z85.030, Z85.038, Z85.04, Z85.040, Z85.048, Z85.05, Z85.06 Z85.060, Z85.068, Z85.07, Z85.09, Z85.1, Z85.11, Z85.110  Z85.118, Z85.12, Z85.2, Z85.20, Z85.21, Z85.22, Z85.23, Z85.230  Z85.238, Z85.29, Z85.3, Z85.4, Z85.40, Z85.41, Z85.42, Z85.43  Z85.44, Z85.45, Z85.46, Z85.47, Z85.48, Z85.49, Z85.5, Z85.50  Z85.51, Z85.52, Z85.520, Z85.528, Z85.53, Z85.54, Z85.59, Z85.6  Z85.7, Z85.71, Z85.72, Z85.79, Z85.8, Z85.81, Z85.810, Z85.818  Z85.819, Z85.82, Z85.820, Z85.821, Z85.828, Z85.83, Z85.830  Z85.831, Z85.84, Z85.840, Z85.841, Z85.848, Z85.85, Z85.850  Z85.858, Z85.89, Z85.9 |
| AIDS | Elixhauser comorbidity |
| Coagulopathy | Elixhauser comorbidity |
| Solid tumor without metastasis | Elixhauser comorbidity |
| Drug abuse | Elixhauser comorbidity |
| Alcohol abuse | Elixhauser comorbidity |
| Nicotine dependence | Elixhauser comorbidity |
| Deficiency anemia | Elixhauser comorbidity |
| Hypothyroidism | Elixhauser comorbidity |
| Previous PCI | Z95.5, Z98.61 |
| Previous CABG | Z95.1, I25.7, I25.70, I25.700, I25.701, I25.702, I25.708, I25.709  I25.71, I25.710, I25.711, I25.712, I25.718, I25.719, I25.72  I25.720, I25.721, I25.722, I25.728, I25.729, I25.73, I25.730  I25.731, I25.732, I25.738, I25.739, I25.810, I25.812 |
| Previous MI | I25.2, I22.0, I22.1, I22.2, I22.8, I22.9 |
| Cerebrovascular disease | I60, I60.00, I60.01, I60.02, I60.10, I60.11, I60.12, I60.2, I60.30  I60.31, I60.32, I60.4, I60.50, I60.51, I60.52, I60.6, I60.7, I60.8,  I60.9, I61, I61.0, I61.1, I61.2, I61.3, I61.4, I61.5, I61.6, I61.8  I61.9, I62, I62.00, I62.01, I62.02, I62.03, I62.1, I62.9, I63, I63.00  I63.011, I63.012, I63.013, I63.019, I63.02, I63.031, I63.032  I63.033, I63.039, I63.09, I63.10, I63.111, I63.112, I63.113, I63.119, I63.12, I63.131, I63.132, I63.133, I63.139, I63.19, I63.20  I63.211, I63.212, I63.213, I63.219, I63.22, I63.231, I63.232, I63.233, I63.239, I63.29, I63.30, I63.311, I63.312, I63.313  I63.319, I63.321, I63.322, I63.323, I63.329, I63.331, I63.332  I63.333, I63.339, I63.341, I63.342, I63.343, I63.349, I63.39  I63.40, I63.411, I63.412, I63.413, I63.419, I63.421, I63.422  I63.423, I63.429, I63.431, I63.432, I63.433, I63.439, I63.441  I63.442, I63.443, I63.449, I63.349, I63.50, I63.511, I63.512  I63.513, I63.519, I63.521, I63.522, I63.523, I63.529, I63.531  I63.532, I63.533, I63.539, I63.541, I63.542, I63.543, I63.549  I63.59, I63.6, I63.8, I63.9, I67.82, I69.00, I69.010, I69.011  I69.012, I69.013, I69.014, I69.015, I69.018, I69.019, I69.020  I69.021, I69.022, I69.023, I69.028, I69.031, I69.032, I69.033  I69.034, I69.039, I69.041, I69.042, I69.043, I69.044, I69.049  I69.051, I69.052, I69.053, I69.054, I69.059, I69.061, I69.062  I69.063, I69.064, I69.065, I69.069, I69.090, I69.092, I69.093  I69.098, I69.10, I69.110, I69.111, I69.112, I69.113, I69.114, I69.115, I69.118, I69.119, I69.120, I69.121, I69.122, I69.123  I69.128, I69.131, I69.132, I69.133, I69.134, I69.139, I69.141  I69.142, I69.143, I69.144, I69.149, I69.151, I69.152, I69.153, I69.154, I69.59, I69.161, I69.162, I69.163, I69.164, I69.165, I69.169, I69.190, I69.192, I69.193, I69.198, I69.20, I69.210, I69.211, I69.212, I69.213, I69.214, I69.215, I69.218, I69.219  I69.220, I69.221, I69.222, I69.223, I69.228, I69.231, I69.232  I69.233, I69.234, I69.239, I69.241, I69.242, I69.243, I69.244  I69.249, I69.251, I69.252, I69.253, I69.254, I69.259, I69.261, I69.262, I69.263, I69.264, I69.265, I69.269, I69.290, I69.292  I69.293, I69.298, I69.30, I69.310, I69.311, I69.312, I69.313  I69.314, I69.315, I69.318, I69.319, I69.320, I69.321, I69.322  I69.323, I69.328, I69.331, I69.332, I69.333, I69.334, I69.339  I69.341, I69.342, I69.343, I69.344, I69.349, I69.351, I69.352  I69.353, I69.354, I69.359, I69.361, I69.362, I69.363, I69.364  I69.365, I69.369, I69.390, I69.392, I69.393, I69.398, I69.80, I69.810, I69.811, I69.812, I69.813, I69.814, I69.815, I69.818  I69.819, I69.820, I69.821, I69.822, I69.823, I69.828, I69.831, I69.832, I69.833, I69.834, I69.839, I69.841, I69.842, I69.843, I69.844, I69.849, I69.851, I69.852, I69.853, I69.854, I69.859  I69.861, I69.862, I69.863, I69.864, I69.865, I69.869, I69.890  I69.892, I69.893, I69.898, I69.90, I69.910, I69.911, I69.912  I69.913, I69.914, I69.915, I69.918, I69.919, I69.920, I69.921  I69.922, I69.923, I69.928, I69.931, I69.932, I69.933, I69.934  I69.939, I69.941, I69.942, I69.943, I69.944, I69.949, I69.951  I69.952, I69.953, I69.954, I69.959, I69.961, I69.962, I69.963  I69.964, I69.969, I69.990, I69.992, I69.993, I69.998, G45.1, G46.0, G46.1, G46.2, G46.3, G46.4, G46.5, G46.6, G46.7, G46.8, Z86.73 |
| Cardiac arrest | I46.9, I46.2, I46.8 |
| Complete atrioventricular block | I44.2 |
| Cardiac tamponade | I31.4 |
| Pericarditis | I30.0, I30.1, I30.8, I30.9 |
| Acute Kidney Injury/Failure | N17.0, N17.1, N17.2, N17.8, N17.9, N99.0 |
| Acute Post Hemorrhagic Anemia | D62 |
| Invasive mechanical ventilation | 5A1935Z, 5A1945Z, 5A1955Z, 0BH17EZ, 0BH18EZ |
| Percutaneous left ventricular assist devices | 5A0221D, 5A0211D, 02HA3RJ, 02HA4RJ, 5A02116, 5A02216  02HA3QZ, 02HA3RS, 02HA3RZ, 02HA4QZ, 02HA4RJ, 02HA4RS, 02HA4RZ |
| Extracorporeal Membrane Oxygenation | 5A1522F, 5A1522G, 5A15A2F, 5A15A2G, 5A15223 |
| Intra-Aortic Balloon Pump | 5A02210 |
| Durable Left Ventricular Assist Devices | 02HA0QZ |
| Renal Replacement Therapy | 5A1D70Z, 5A1D80Z, 5A1D90Z, 5A1D60Z, 5A1D00Z |
| Encounter for Palliative Care | Z51.5 |
| Do Not Resuscitate | Z66 |
| Percutaneous Coronary Intervention | 027034, 0270346, 027034Z, 027035, 0270356, 027035Z, 027036 , 0270366, 027036Z, 027037, 0270376, 027037Z, 027044, 0270446, 027044Z, 027045, 0270456, 027045Z, 027046, 0270466, 027046Z, 027047, 0270476, 027047Z, 0271346  027134Z, 027135, 0271356, 027135Z, 027136, 0271366, 027136Z, 027137, 0271376, 027137Z, 027144, 0271446, 027144Z, 0271456, 027145Z, 0271466, 027146Z, 0271476  027147Z, 027234, 0272346, 027234Z, 027235, 0272356, 027235Z  027236, 0272366, 027236Z, 027237, 0272376, 027237Z, 027244, 0272446, 027244Z, 027245, 0272456, 027245Z, 027246, 0272466  027246Z, 027247, 0272476, 027247Z, 027334, 0273346, 027334Z, 0273356, 027335Z, 0273366, 027336Z, 0273376, 027337Z, 027344, 0273446, 027344Z, 0273456, 027345Z, 0273466, 027346Z, 0273476, 027347Z, 02703D, 02703D6, 02703DZ, 02703E, 02703E6, 02703EZ, 02703F, 02703F6, 02703FZ, 02703G, 02703G6, 02703GZ, 02704D, 02704D6, 02704DZ, 02704E, 02704E6, 02704EZ, 02704F, 02704F6, 02704FZ, 02704G, 02704G6, 02704GZ, 02713D6, 02713DZ, 02713E, 02713E6, 02713EZ, 02713F, 02713F6, 02713FZ, 02713G, 02713G6, 02713GZ, 02714D, 02714D6, 02714DZ, 02714E, 02714E6, 02714EZ, 02714F, 02714F6, 02714FZ , 02714G, 02714G6, 02714GZ, 02723D, 02723D6, 02723DZ, 02723E, 02723E6, 02723EZ, 02723F, 02723F6, 02723FZ, 02723G, 02723G6, 02723GZ, 02724D, 02724D6, 02724DZ, 02724E, 02724E6, 02724EZ, 02724F, 02724F6, 02724FZ, 02724G, 02724G6, 02724GZ, 02733D, 02733D6, 02733DZ  02733E, 02733E6, 02733EZ, 02733F, 02733F6, 02733FZ, 02733G, 02733G6, 02733GZ, 02734D, 02734D6, 02734DZ, 02734E, 02734E6, 02734EZ, 02734F, 02734F6, 02734FZ, 02734G, 02734G6, 02734GZ, 02703Z, 02703Z6, 02703ZZ, 02704Z6, 02704ZZ, 02713Z, 02713Z6, 02713ZZ, 02714Z, 02714Z6, 02714ZZ, 02723Z, 02723Z6, 02723ZZ, 02724Z, 02724Z6, 02724ZZ, 02733Z, 02733Z6, 02733ZZ, 02734Z, 02734Z6, 02734ZZ |
| Coronary Artery Bypass Graft | 02130KW, 02130Z3, 02130Z8, 02130Z9, 02130ZC, 02130ZF, 02130K8, 02130K9, 02130KC, 02130KF, 02130A9, 02130AC, 02130AF, 02130AW, 02130J3, 02130J8, 02130J9, 02130JC, 02130JF, 02130JW, 02130K3, 02120Z8, 02120Z9, 02120ZC, 02120ZF, 0213093, 0213098, 0213099, 021309C, 021309F, 021309W, 02130A3, 02130A8, 02120AW, 02120J3, 02120J8, 02120J9, 02120JC, 02120JF, 02120JW, 02120K3, 02120K8, 02120K9, 02120KC, 02120KF, 02120KW, 02120Z3, 02110Z9  02110ZC, 02110ZF, 0212093, 0212098, 0212099, 021209C  021209F, 021209W, 02120A3, 02120A8, 02120A9, 02120AC,  02120AF, 02110J3, 02110J8, 02110J9, 02110JC, 02110JF, 02110JW, 02110K3, 02110K8, 02110K9, 02110KC, 02110KF  02110KW, 02110Z3, 02110Z8, 02100ZC, 02100ZF, 0211093  0211098, 0211099, 021109C, 021109F, 021109W, 02110A3  02110A8, 02110A9, 02110AC, 02110AF, 02110AW, 02100J3  02100K9, 02100KC, 02100KF, 02100KW, 02100Z3, 02100Z8, 02100Z9, 0210093, 0210099, 021009C, 021009F, 021009W, 02100A3, 02100A8, 02100A9, 02100AC, 02100AF, 02100AW |
| Gastrostomy | 0D16074, 0D160J4, 0D160K4, 0D163J4, 0D16474, 0D164J4  0D164K4, 0D164Z4, 0D160Z4, 0D16874, 0D168J4, 0D168K4  0D168Z4, 0D9600Z, 0D960ZZ, 0D9640Z, 0D964ZZ, 0DC60ZZ  0DC63ZZ, 0DC64ZZ, 0DH603Z, 0DH60UZ, 0DH63UZ, 0DH633Z, 0DH64UZ, 0DH643Z, 0DH67UZ, 0DH68UZ, 0DH683Z, 0DH673Z, 0D9630Z, 0D963ZZ |
| Tracheostomy | 0B110F4, 0B110Z4, 0B113F4, 0B113Z4, 0B114F4, 0B114Z4 |
| GI Bleeding | I85.01, I85.11, K25.0, K25.2, K25.4, K25.6, K26.0, K26.2, K26.4  K26.6, K27.0, K27.2, K27.4, K27.6, K28.0, K28.2, K28.4, K28.6, K29.01, K29.21, K29.31, K29.41, K29.51, K29.61, K29.71, K29.81, K29.91, K62.5, K92.0, K92.1, K92.2 |
| Pulmonary embolism | I26, I26.0, I26.01, I26.02, I26.09, I26.9, I26.90, I26.92, I26.93, I26.94, I26.99 |
| Respiratory Failure | J95.1, J95.2, J95.3, J95.82, J95.821, J95.822, J96.00, J96.01, J96.02, J96.20, J96.21, J96.22, J96.90, J96.91, J96.92, R06.03 |

**Supplemental Table 3:** Temporal changes in clinical characteristics of hospitalizations with cardiogenic shock and acute myocardial infarction of patients aged 18-79 years in the United States, 2003-2021.

IQR=Interquartile range; PCI=Percutaneous coronary intervention; CABG=Coronary artery bypass grafting; MI=Myocardial infarction.

n<11 data are not reported (NR) according to HCUP recommendations.

|  | **2003-2006** | **2007-2010** | **2011-2014** | **2016-2019** | **2020-2021** | **Total** | **p-value** |
| --- | --- | --- | --- | --- | --- | --- | --- |
| Sample Size | 102,343 (15.2%) | 120,240 (17.8%) | 154,779 (22.9%) | 195,355 (28.9%) | 102,775 (15.2%) | 675,491 |  |
| Total Hospitalizations with Acute Myocardial Infarction | 2,537,906 (21.7%) | 2,443,246 (20.9%) | 2,530,164 (21.6%) | 2,891,915 (24.7%) | 1,312,799 (11.2%) | 11,713,030 |  |
| Proportion of Cardiogenic Shock among all Hospitalizations with Acute Myocardial Infarction | 4.0% | 4.9% | 6.1% | 6.8% | 7.8% | 5.8% | <0.001 |
| Age (Median, IQR) | 65 (57-72) | 66 (57-73) | 64 (56-72) | 64 (56-71) | 65 (57-72) | 65 (57-72) | <0.001 |
| Sex   1. Female 2. Male | 38,737 (37.9%)  63,605 (62.2%) | 42,187 (35.1%)  78,045 (64.9%) | 52,795 (34.1%)  101,979 (65.9%) | 64,340 (32.9%)  130,990 (67.1%) | 33,640 (32.7%)  69,125 (67.3%) | 231,699 (34.3%)  443,745 (65.7%) | <0.001 |
| Race/ethnicity   1. White 2. Black 3. Hispanic 4. Asian or Pacific Islander 5. Native American/Other | 56,873 (76.6%)  5,671 (7.6%)  6,657 (9.0%)  2,007 (2.7%)  3,006 (4.1%) | 73,123 (74.1%)  8,921 (9.1%)  8,206 (8.3%)  3,243 (3.3%)  5,139 (5.2%) | 103,434 (72.3%)  14,654 (10.2%)  12,780 (8.9%)  4,689 (3.3%)  7,489 (5.2%) | 132,310 (71.0%)  20,105 (10.8%)  18,045 (9.7%)  7,130 (3.8%)  8,815 (4.7%) | 67,905 (69.1%)  10,870 (11.1%)  10,550 (10.7%)  4,065 (4.1%)  4,860 (5.0%) | 433,645 (72.2%)  60,221 (10.0%)  56,238 (9.4%)  21,135 (3.5%)  29,310 (4.9%) | <0.001 |
| Insurance type   1. Medicare 2. Medicaid 3. Private Insurance 4. Self-Pay 5. No Charge/Other | 56,647 (55.4%)  7,332 (7.2%)  29,473 (28.8%)  5,409 (5.3%)  3,387 (3.3%) | 61,605 (51.3%)  10,363 (8.6%)  35,442 (29.5%)  7,881 (6.6%)  4,702 (3.9%) | 82,552 (53.4%)  15,278 (9.9%)  39,997 (25.9%)  10,874 (7.0%)  5,764 (3.7%) | 106,235 (54.5%)  22,390 (11.5%)  49,420 (25.3%)  10,220 (5.2%)  6,755 (3.5%) | 54,650 (53.3%)  13,030 (12.7%)  25,665 (25.0%)  5,140 (5.0%)  4,115 (4.0%) | 361,689 (53.6%)  68,393 (10.1%)  179,997 (26.7%)  39,525 (5.9%)  24,722 (3.7%) | <0.001 |
| Median household income quartile of patient’s ZIP Code   1. 0-25th 2. 26-50th 3. 50-75th 4. 75-100th | 26,583 (26.7%)  26,794 (26.9%)  24,555 (24.7%)  21,658 (21.8%) | 33,216 (28.5%)  31,572 (27.0%)  28,257 (24.2%)  23,708 (20.3%) | 45,623 (30.3%)  40,797 (27.1%)  35,581 (23.6%)  28,762 (19.1%) | 58,845 (30.8%)  51,920 (27.2%)  45,255 (23.7%)  34,940 (18.3%) | 31,340 (31.1%)  27,480 (27.2%)  23,410 (23.2%)  18,635 (18.5%) | 195,607 (29.7%)  178,563 (27.1%)  157,057 (23.8%)  127,702 (19.4%) | <0.001 |
| Hospital location   1. Rural 2. Urban Nonteaching 3. Urban Teaching | 7,075 (6.9%)  43,592 (42.6%)  51,648 (50.5%) | 8,008 (6.7%)  48,674 (40.8%)  62,690 (52.5%) | 7,902 (5.1%)  50,684 (32.8%)  95,756 (62.0%) | 8,480 (4.3%)  35,910 (18.4%)  150,965 (77.3%) | 4,715 (4.6%)  15,145 (14.7%)  82,915 (80.7%) | 36,181 (5.4%)  194,005 (28.8%)  443,973 (65.9%) | <0.001 |
| Region of hospital   1. Northeast 2. Midwest 3. South 4. West | 18,234 (17.8%)  23,576 (23.0%)  40,330 (39.4%)  20,202 (19.7%) | 20,616 (17.2%)  28,314 (23.6%)  44,585 (37.1%)  26,725 (22.2%) | 25,563 (16.5%)  32,822 (21.2%)  63,896 (41.3%)  32,498 (21.0%) | 29,865 (15.3%)  42,345 (21.7%)  81,080 (41.5%)  42,065 (21.5%) | 16,105 (15.7%)  22,310 (21.7%)  42,490 (41.3%)  21,870 (21.3%) | 110,383 (16.3%)  149,366 (22.1%)  272,382 (40.3%)  143,360 (21.2%) | 0.427 |
| **Comorbidities** |  |  |  |  |  |  |  |
| Congestive heart failure | 57,748 (56.4%) | 62,629 (52.1%) | 86,470 (55.9%) | 123,530 (63.2%) | 69,025 (67.2%) | 399,401 (59.1%) | <0.001 |
| Valvular disease | 2,773 (2.8%) | 2,494 (2.8%) | 5,527 (3.6%) | 35,605 (18.2%) | 18,335 (17.8%) | 64,734 (10.1%) | <0.001 |
| Chronic pulmonary disease | 24,638 (24.6%) | 21,585 (24.2%) | 37,698 (24.4%) | 35,465 (18.2%) | 7,735 (7.5%) | 127,121 (19.8%) | <0.001 |
| Obesity | 5,028 (5.0%) | 8,812 (9.9%) | 24,640 (15.9%) | 28,205 (14.4%) | 6,990 (6.8%) | 73,676 (11.5%) | <0.001 |
| Dementia | 591 (0.6%) | 966 (0.8%) | 1,269 (0.8%) | 5,065 (2.6%) | 2,670 (2.6%) | 10,561 (1.6%) | <0.001 |
| Dyslipidemia | 19,060 (18.6%) | 40,337 (33.6%) | 70,503 (45.6%) | 99,695 (51.0%) | 54,795 (53.3%) | 284,389 (42.1%) | <0.001 |
| Iron deficiency | 2,522 (2.5%) | 3,002 (2.5%) | 4,519 (2.9%) | 7,070 (3.6%) | 3,780 (3.7%) | 20,893 (3.1%) | <0.001 |
| Diabetes Mellitus | 26,730 (26.6%) | 29,848 (33.4%) | 61,338 (39.6%) | 67,115 (34.4%) | 16,290 (15.9%) | 201,321 (31.3%) | <0.001 |
| Hypertension | 38,035 (37.9%) | 44,129 (49.4%) | 92,456 (59.7%) | 119,535 (61.2%) | 28,650 (27.9%) | 322,805 (50.2%) | <0.001 |
| Liver disease | 1,419 (1.4%) | 1,670 (1.9%) | 4,811 (3.1%) | 12,950 (6.6%) | 7,950 (7.7%) | 28,800 (4.5%) | <0.001 |
| Neurological disorders | 4,969 (5.0%) | 5,590 (6.3%) | 12,105 (7.8%) | 37,475 (19.2%) | 23,855 (23.2%) | 83,995 (13.1%) | <0.001 |
| Peripheral vascular disease | 7,141 (7.1%) | 10,914 (12.2%) | 23,671 (15.3%) | 19,570 (10.0%) | 3,585 (3.5%) | 64,881 (10.1%) | <0.001 |
| Renal failure | 13,712 (13.7%) | 17,173 (19.2%) | 37,079 (24.0%) | 53,780 (27.5%) | 28,585 (27.8%) | 150,330 (23.4%) | <0.001 |
| Malnutrition | 3,280 (3.2%) | 8,098 (6.7%) | 14,913 (9.6%) | 18,195 (9.3%) | 9,235 (9.0%) | 53,722 (8.0%) | <0.001 |
| Atrial fibrillation | 22,688 (22.2%) | 22,816 (19.0%) | 36,180 (23.4%) | 54,450 (27.9%) | 29,085 (28.3%) | 165,219 (24.5%) | <0.001 |
| History of malignancy | 2,244 (2.2%) | 4,030 (3.4%) | 7,249 (4.7%) | 11,680 (6.0%) | 5,525 (5.4%) | 30,728 (4.6%) | <0.001 |
| Coagulopathy | 11,690 (11.7%) | 12,416 (13.9%) | 30,325 (19.6%) | 44,820 (22.9%) | 23,820 (23.2%) | 123,071 (19.2%) | <0.001 |
| Solid tumor without metastasis | 1,374 (1.4%) | 1,505 (1.7%) | 2,751 (1.8%) | 3,175 (1.6%) | 795 (0.8%) | 9,600 (1.5%) | <0.001 |
| Drug abuse | 1,388 (1.4%) | 1,988 (2.2%) | 4,939 (3.2%) | 5,390 (2.8%) | 1,255 (1.2%) | 14,960 (2.3%) | <0.001 |
| Alcohol abuse | 3,286 (3.3%) | 3,967 (4.4%) | 8,428 (5.5%) | 7,570 (3.9%) | 1,625 (1.6%) | 24,876 (3.9%) | <0.001 |
| Nicotine dependence | 15,548 (15.2%) | 31,498 (26.2%) | 54,966 (35.5%) | 47,895 (24.5%) | 23,275 (22.7%) | 173,182 (25.6%) | <0.001 |
| Deficiency anemia | 10,020 (10.0%) | 16,055 (18.0%) | 33,458 (21.6%) | 44,255 (22.7%) | 23,145 (22.5%) | 126,933 (19.8%) | <0.001 |
| Hypothyroidism | 4,029 (4.0%) | 5,263 (5.9%) | 12,803 (8.3%) | 12,740 (6.5%) | 2,800 (2.7%) | 37,636 (5.9%) | <0.001 |
| Previous PCI | 3,608 (3.5%) | 8,347 (6.9%) | 16,048 (10.4%) | 26,060 (13.3%) | 11,340 (11.0%) | 65,403 (9.7%) | <0.001 |
| Previous CABG | 5,875 (5.7%) | 9,556 (8.0%) | 14,100 (9.1%) | 16,775 (8.6%) | 7,815 (7.6%) | 54,121 (8.0%) | <0.001 |
| Previous MI | 4,535 (4.4%) | 8,889 (7.4%) | 15,252 (9.9%) | 24,900 (12.8%) | 13,225 (12.9%) | 66,802 (9.9%) | <0.001 |
| Cerebrovascular disease | 4,650 (4.5%) | 9,537 (7.9%) | 16,659 (10.8%) | 25,290 (13.0%) | 12,600 (12.3%) | 68,736 (10.2%) | <0.001 |
| **Type of Myocardial Infarction** | | | | | | | |
| STEMI | 72,111 (70.5%) | 75,096 (62.5%) | 83,821 (54.2%) | 97,220 (49.8%) | 50,750 (49.4%) | 378,998 (56.1%) | <0.001 |
| NSTEMI | 30,232 (29.5%) | 45,143 (37.5%) | 70,958 (45.8%) | 98,135 (50.2%) | 52,025 (50.6%) | 296,494 (43.9%) | <0.001 |

**Supplemental Table 4:** Outcomes of hospitalizations with cardiogenic shock with acute myocardial infarction of patients aged 18-79 years in the United States, 2003-2021. P values for all outcomes are derived from multivariable regression analysis.

Variables included in the multivariable regression model: Time period, Sex, Race/ethnicity, Insurance type, ZIP Code income quartile, Hospital location, Region of Hospital, Congestive heart failure, Valvular disease, Chronic pulmonary disease, Obesity, Dementia, Dyslipidemia, Diabetes mellitus, Hypertension, Liver disease, Peripheral vascular disease, Renal failure, Malnutrition, Atrial fibrillation, History of malignancy, Coagulopathy, Solid tumor without metastasis, Alcohol abuse, Nicotine dependence, Deficiency anemia, Hypothyroidism, Previous PCI, Previous CABG, Previous MI, Cerebrovascular disease, STEMI

*n<11 data are not reported (NR) according to HCUP recommendations. In some cases, additional suppression is applied to prevent deductive disclosure.

|  | **2003-2006** | **2007-2010** | **2011-2014** | **2016-2019** | **2020-2021** | **Total** | **p-value** |
| --- | --- | --- | --- | --- | --- | --- | --- |
| Sample Size | 102,343 (15.2%) | 120,240 (17.8%) | 154,779 (22.9%) | 195,355 (28.9%) | 102,775 (15.2%) | 675,491 |  |
| Cardiac Arrest | 16,638 (16.3%) | 23,190 (19.3%) | 38,065 (24.6%) | 21,890 (11.2%) | 19,555 (19.0%) | 119,339 (17.7%) | <0.001 |
| Cardiac Tamponade | NR* | 1,209 (1.0%) | 1,631 (1.1%) | 2,625 (1.3%) | 1,410 (1.4%) | NR* | - |
| Pericarditis | 578 (0.6%) | 500 (0.4%) | 500 (0.3%) | 675 (0.4%) | 365 (0.4%) | 2,618 (0.4%) | 0.015 |
| Acute Kidney Injury | 30,347 (29.7%) | 48,020 (39.9%) | 74,106 (47.9%) | 105,540 (54.0%) | 59,045 (57.5%) | 317,058 (46.9%) | <0.001 |
| Acute Post Hemorrhagic Anemia | 4,754 (4.7%) | 10,209 (8.5%) | 23,529 (15.2%) | 41,285 (21.1%) | 23,615 (23.0%) | 103,392 (15.3%) | <0.001 |
| Invasive Mechanical Ventilation | 49,626 (48.5%) | 63,773 (53.0%) | 87,203 (56.3%) | 105,655 (54.1%) | 52,965 (51.5%) | 359,223 (53.2%) | <0.001 |
| Mechanical Circulatory Support   1. Percutaneous Left Ventricular Assist Devices 2. Extracorporeal Membrane Oxygenation 3. Intra-Aortic Balloon Pump | 45,167 (44.1%)  57 (0.1%)  194 (0.2%)  45,072 (44.0%) | 55,186 (45.9%)  828 (0.7%)  704 (0.6%)  54,473 (45.3%) | 62,942 (40.7%)  5,569 (3.6%)  2,443 (1.6%)  58,258 (37.6%) | 77,530 (39.7%)  24,425 (12.5%)  6,710 (3.4%)  54,285 (27.8%) | 39,235 (38.2%)  14,970 (14.6%)  3,425 (3.3%)  25,425 (24.7%) | 280,061 (41.5%)  45,849 (6.8%)  13,476 (2.0%)  237,513 (35.2%) | <0.001  <0.001  <0.001  <0.001 |
| Renal Replacement Therapy | 6,117 (6.0%) | 9,194 (7.7%) | 14,878 (9.6%) | 21,755 (11.1%) | 12,530 (12.2%) | 64,473 (9.5%) | 0.184 |
| Encounter for Palliative Care | 719 (0.7%) | 4,156 (3.5%) | 13,823 (8.9%) | 26,515 (13.6%) | 16,255 (15.8%) | 61,468 (9.1%) | <0.001 |
| Do Not Resuscitate | NR* | 358 (0.3%) | 16,710 (10.8%) | 35,495 (18.2%) | 21,375 (20.8%) | NR* | - |
| Percutaneous Coronary Intervention | 37,745 (36.9%) | 54,416 (45.3%) | 68,610 (44.3%) | 86,140 (44.1%) | 42,800 (41.6%) | 289,712 (42.9%) | <0.001 |
| Coronary Artery Bypass Graft | 18,985 (18.6%) | 22,094 (18.4%) | 26,254 (17.0%) | 34,115 (17.5%) | 17,720 (17.2%) | 119,168 (17.6%) | <0.001 |
| Gastrostomy | 2,196 (2.2%) | 2,923 (2.4%) | 3,842 (2.5%) | 7,370 (3.8%) | 3,815 (3.7%) | 20,146 (3.0%) | <0.001 |
| Tracheostomy | 4,884 (4.8%) | 5,692 (4.7%) | 6,805 (4.4%) | 6,580 (3.4%) | 3,305 (3.2%) | 27,267 (4.0%) | <0.001 |
| Gastrointestinal Bleeding | 6,933 (6.8%) | 8,217 (6.8%) | 10,487 (6.8%) | 13,750 (7.0%) | 7,200 (7.0%) | 46,587 (6.9%) | 0.174 |
| Pulmonary Embolism | 1,106 (1.1%) | 1,721 (1.4%) | 2,874 (1.9%) | 4,105 (2.1%) | 2,490 (2.4%) | 12,297 (1.8%) | <0.001 |
| Respiratory Failure | 45,056 (44.0%) | 61,442 (51.1%) | 99,637 (64.4%) | 130,615 (66.9%) | 68,030 (66.2%) | 404,779 (59.9%) | <0.001 |
| In-hospital Mortality | 40,052 (39.2%) | 38,791 (32.3%) | 50,374 (32.6%) | 60,930 (31.2%) | 32,580 (31.7%) | 222,727 (33.0%) | <0.001 |
| Length of Stay | 6.8 (2.5-13.5) | 7.0 (2.7-13.7) | 6.9 (2.7-13.2) | 6.6 (2.5-12.9) | 6.6 (2.4-13.0) | 6.8 (2.5-13.2) | <0.001 |
| Total Hospitalization Cost (USD 2021) | 108,258 (55.389-200,245) | 128,188 (68,058-238,615) | 147,938 (79,774-268,972) | 183,075 (97,091-336,630) | 196,338 (102,613-369,619) | 153,138 (79,627-286,847) | <0.001 |
| Discharge Disposition   1. Routine Transfer to Home or Self-Care 2. Transfer to Short-term Hospital 3. Transfer to Facility (Skilled Nursing Facility, Intermediate Care Facility, etc.) 4. Died 5. Home Health Care/Other | 28,105 (27.5%)  7,956 (7.8%)  15,525 (15.2%)  40,052 (39.2%)  10,444 (10.2%) | 34,296 (28.5%)  9,482 (7.9%)  23,233 (19.3%)  38,791 (32.3%)  14,374 (12.0%) | 42,643 (27.6%)  11,974 (7.7%)  30,628 (19.8%)  50,374 (32.6%)  19,062 (12.3%) | 51,950 (26.6%)  16,460 (8.4%)  40,400 (20.7%)  60,930 (31.2%)  25,490 (13.1%) | 26,340 (25.6%)  8,880 (8.6%)  18,635 (18.1%)  32,580 (31.7%)  16,330 (15.9%) | 183,333 (27.2%)  54,752 (8.1%)  128,421 (19.0%)  222,727 (33.0%)  85,700 (12.7%) | <0.001 |

**Supplemental Table 5:** Outcomes of hospitalizations with cardiogenic shock with acute myocardial infarction of patients aged 18 - 79 years old stratified by STEMI vs NSTEMI, 2003-2021. P values for all outcomes are derived from multivariable regression analysis.

Variables included in the multivariable regression model: Time period, Sex, Race/ethnicity, Insurance type, ZIP Code income quartile, Hospital location, Region of Hospital, Congestive heart failure, Valvular disease, Chronic pulmonary disease, Obesity, Dementia, Dyslipidemia, Diabetes mellitus, Hypertension, Liver disease, Peripheral vascular disease, Renal failure, Malnutrition, Atrial fibrillation, History of malignancy, Coagulopathy, Solid tumor without metastasis, Alcohol abuse, Nicotine dependence, Deficiency anemia, Hypothyroidism, Previous PCI, Previous CABG, Previous MI, Cerebrovascular disease, STEMI

|  | **STEMI** | **NSTEMI** | **p-value** |
| --- | --- | --- | --- |
| Sample Size | 378,998 (56.1%) | 296,494 (43.9%) |  |
| Cardiac Arrest | 76,113 (20.1%) | 43,227 (14.6%) | <0.001 |
| Cardiac Tamponade | 3,997 (1.1%) | 2,878 (1.0%) | 0.070 |
| Pericarditis | 1,651 (0.4%) | 967 (0.3%) | 0.210 |
| Acute Kidney Injury | 152,524 (40.2%) | 164,534 (55.5%) | <0.001 |
| Acute Post Hemorrhagic Anemia | 46,121 (12.2%) | 57,271 (19.3%) | <0.001 |
| Invasive Mechanical Ventilation | 196,326 (51.8%) | 162,897 (55.0%) | 0.838 |
| Mechanical Circulatory Support   1. Percutaneous Left Ventricular Assist Devices 2. Extracorporeal Membrane Oxygenation 3. Intra-Aortic Balloon Pump | 190,119 (50.2%)  30,038 (7.9%)  8,914 (2.4%)  162,297 (42.8%) | 89,941 (30.3%)  15,811 (5.3%)  4,562 (1.5%)  75,216 (25.4%) | <0.001  <0.001  <0.001  <0.001 |
| Renal Replacement Therapy | 24,341 (6.4%) | 40,132 (13.5%) | <0.001 |
| Encounter for Palliative Care | 29,740 (7.9%) | 31,728 (10.7%) | <0.001 |
| Do Not Resuscitate | 35,359 (9.3%) | 38,580 (13.0%) | <0.001 |
| Percutaneous Coronary Intervention | 223,619 (59.0%) | 66,093 (22.3%) | <0.001 |
| Coronary Artery Bypass Graft | 53,018 (14.0%) | 66,150 (22.3%) | <0.001 |
| Gastrostomy | 9,327 (2.5%) | 10,819 (3.7%) | 0.002 |
| Tracheostomy | 13,885 (3.7%) | 13,381 (4.5%) | 0.975 |
| Gastrointestinal Bleeding | 25,363 (6.7%) | 21,224 (7.2%) | 0.309 |
| Pulmonary Embolism | 5,049 (1.3%) | 7,249 (2.4%) | <0.001 |
| Respiratory Failure | 210,053 (55.4%) | 194,726 (65.7%) | <0.001 |
| In-hospital Mortality | 128,879 (34.0%) | 93,848 (31.7%) | <0.001 |
| Length of Stay | 5.5 (1.9-11.4) | 8.5 (3.8-15.2) | <0.001 |
| Total Hospitalization Cost (USD 2021) | 143,627 (78,794-265,203) | 167,271 (81,124-315,098) | <0.001 |
| Discharge Disposition   1. Routine Transfer to Home or Self-Care 2. Transfer to Short-term Hospital 3. Transfer to Facility (Skilled Nursing Facility, Intermediate Care Facility, etc.) 4. Died 5. Home Health Care/Other | 121,461 (32.1%)  29,817 (7.9%)  57,210 (15.1%)  128,879 (34.0%)  41,302 (10.9%) | 61,873 (20.9%)  24,935 (8.4%)  71,211 (24.0%)  93,848 (31.7%)  44,398 (15.0%) | <0.001 |

**Supplemental Figure 1:** In-hospital mortality in hospitalizations with cardiogenic shock and acute myocardial infarction in adults (A) >= 80 years and (B) 18-79 years in the United States from 2003 to 2021 stratified by United States region

(A)

(B)
